# Data supporting a molecular phylogeny of the hyper-diverse genus *Brueelia*

**DOI:** 10.1016/j.dib.2015.10.022

**Published:** 2015-11-02

**Authors:** Sarah E. Bush, Jason D. Weckstein, Daniel R. Gustafsson, Julie Allen, Emily DiBlasi, Scott M. Shreve, Rachel Boldt, Heather R. Skeen, Kevin P. Johnson

**Affiliations:** a Department of Biology, University of Utah, 257 South 1400 East, Salt Lake City, UT 84112, USA; b Field Museum of Natural History, Science and Education, Integrative Research Center, 1400 S. Lake Shore Drive, Chicago, IL 60605, USA; c Illinois Natural History Survey, University of Illinois, 1816 South Oak Street, Champaign, IL 61820, USA

**Keywords:** Brueelia, Lice, Songbirds, Host-specificity, Phylogenetic reconstruction, Macroevolution

## Abstract

Data is presented in support of a phylogenetic reconstruction of one of the largest, and most poorly understood, groups of lice: the *Brueelia*-complex (Bush et al., 2015[1]). Presented data include the voucher information and molecular data (GenBank accession numbers) of 333 ingroup taxa within the *Brueelia-*complex and 30 outgroup taxa selected from across the order Phthiraptera. Also included are phylogenetic reconstructions based on Bayesian inference analyses of combined COI and EF-1α sequences for *Brueelia*-complex species and outgroup taxa.

## Specifications table

**Table:** 

Subject area	Biology, genetics and genomics
More specific subject area	Phylogenetics
Type of data	Specimen matrix, phylogenetic reconstruction
How data was acquired	Phylogenetic reconstruction using Bayesian inference methods
Data format	Raw, analyzed
Experimental factors	n/a
Experimental features	n/a
Data source location	worldwide
Data accessibility	Within this article, sequences available in GenBank

## Value of the data

• Evolutionary history of feather lice in the Brueelia-complex was reconstructed*.*
• Data support re-recognition of historic genera, and erection of several new genera.
• Associations of lice with geography and host-family are correlated with phylogeny*.*
• Host association and geographic origin of each sequenced specimen are provided*.*

## 1 Data, materials and methods

The data presented herein supports a phylogenetic reconstruction of the *Brueelia*-complex; these data complement the companion article by Bush et al. [1].

### 1.1 Sampling

We sampled a total of 333 louse specimens belonging to the *Brueelia*-complex (Supplemental Table 1). These lice were sampled from 250 bird species belonging to 66 bird families and five orders (Passeriformes, Coraciiformes, Cuculiformes, Piciformes, and Trogoniformes). Sampled lice include 38 known species and 211 lice that represent either new species of lice or new host associations. These samples were collected from 23 countries and all continents except Antarctica. An additional 30 outgroup taxa for rooting the phylogeny were selected to represent nested sister taxonomic relationships within the family Philopteridae [2], [3]. These 30 louse outgroup species were from 27 host species, in 17 host families, collected from 9 countries.

Lice were collected either from euthanized bird specimens using ethyl acetate fumigation or from live birds dusted with pyrethrum powder [4], [5]. Care was taken to make sure that individual hosts were kept separate at all times and to clean all working surfaces between fumigation. Lice were collected by the authors and colleagues during field-work conducted over several decades and were stored in vials of 95% ethanol, usually in ultracold (−80 °C) freezers.

### 1.2 DNA extraction, amplification and alignment

DNA was extracted from lice using either the Qiagen DNeasy micro-kit (Valencia, California, USA) following the manufacturer׳s protocol as described by Valim and Weckstein [6], or the Qiagen DNeasy tissue kit (Valencia, California, USA) following the manufacture׳s protocol as described by Johnson et al. [7]. After DNA was extracted from individual lice, the exoskeletons were retained and mounted on microscope slides [8]. These voucher slides were used to identify each specimen to genus using the keys in Price et al. [9]. Specific-level identifications were based on original descriptions, specific keys if possible, and comparison with identified slide mounted material. Voucher slides are deposited in the Illinois Natural History Survey Insect Collection (INHS), Price Institute for Parasite Research at the University of Utah (PIPeR), and Field Museum of Natural History (FMNH) (Supplemental Table 1).

Portions of one mitochondrial (COI) and one nuclear gene (EF-1α) were selected because these genes have successfully resolved phylogenies of closely related groups of parasitic lice and more distantly related “bark lice” [3], [10], [11], [12], [13]. We used PCR to amplify and sequence portions of the mitochondrial cytochrome oxidase I (COI; 379 bp) and the nuclear gene elongation factor 1a (EF1 α; 347 bp) using published amplification and sequencing protocols [12], [13]. Purified PCR products were cycle sequenced using ABI Big Dye (Applied Biosystems, Foster City, California) and run on an ABI Prism 3730 DNA sequencer (Applied Biosystems). Raw sequence data were trimmed, edited, and reconciled using Sequencher 5.0.1 (Genecodes CO., Ann Arbor, Michigan) or Geneious (version 7.0.3, Biomatters LTD). Both genes are protein coding and therefore we were able to easily align them by eye according to codons. These aligned gene sequences were then concatenated for phylogenetic analysis.

### 1.3 Phylogenetic analyses

The final sequence alignment was analyzed using PartitionFinder (v1.1.1; [14], [15]), an open source python script that selects the best-fit partitioning schemes and models of molecular evolution for phylogenetic analysis. We tested whether the two genes (COI, EF1 α) should be analyzed together under the same model and parameters or as two separate partitions. We tested only these two partitions because separating each of these genes by codon would only provide 100 bps for each partition, a very small amount of sequence for estimating parameters and would likely result in over-parameterization. The PartitionFinder analysis found that a single partition and GTR+I+G model of molecular evolution best fit the data, using both AICc and BIC criterion. Using these parameters, which were estimated as part of the analysis, and a flat Dirichlet prior for state frequencies, we ran a Bayesian analysis in MrBayes 3.2.2 [16], [17], [18] for 10,000,000 generations. Each Bayesian analysis included two parallel runs, each with four Markov chains, to ensure that our analyses were not stuck at local optima [19]. Markov chains were sampled every 500 generations, yielding 20,000 parameter point-estimates. We used these 20,000 point-estimates minus the burn-in generations (500 point-estimates, 250,000 generations) to create a 50% majority-rule consensus tree and calculated Bayesian posterior probabilities to assess nodal support. We rooted the Bayesian tree using a nested set of sister taxa within the family Philopteridae [2], [12], [13], [20].

**Fig. 1:**
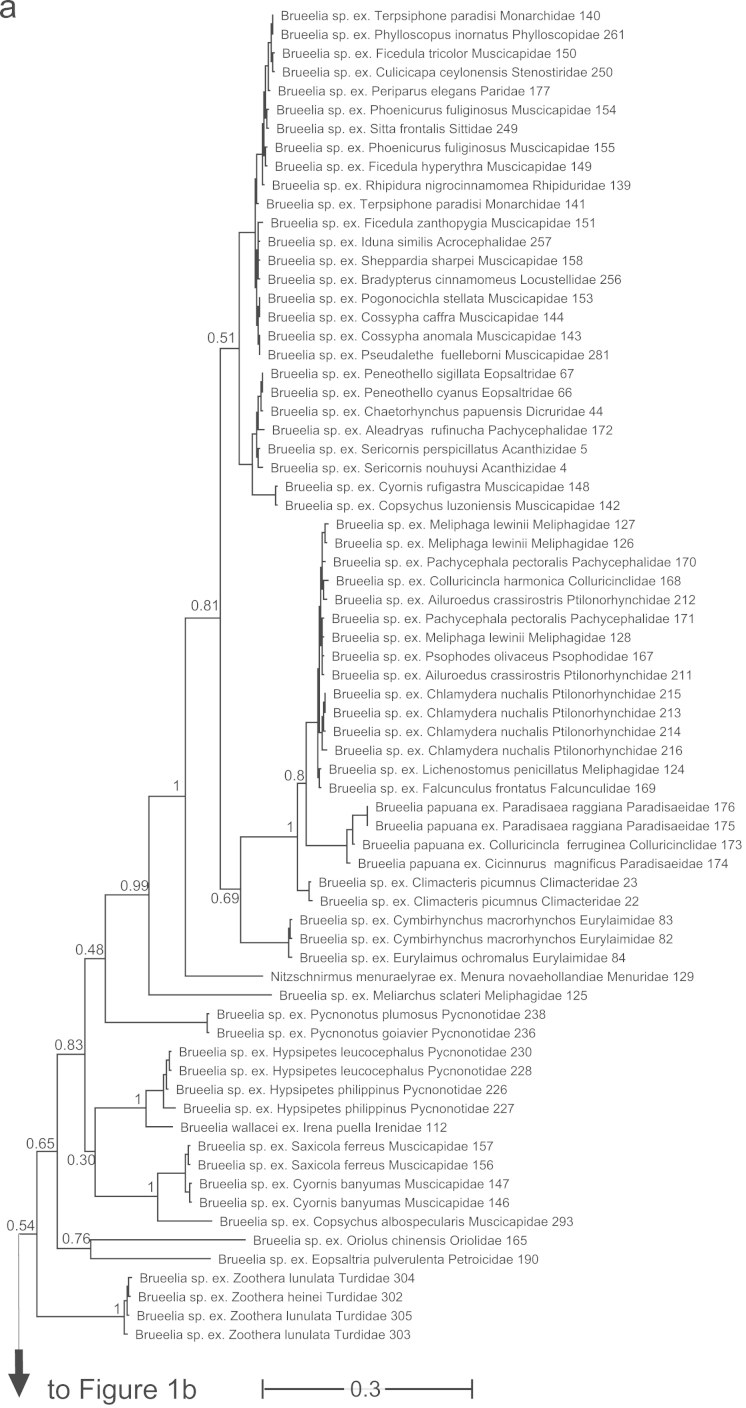

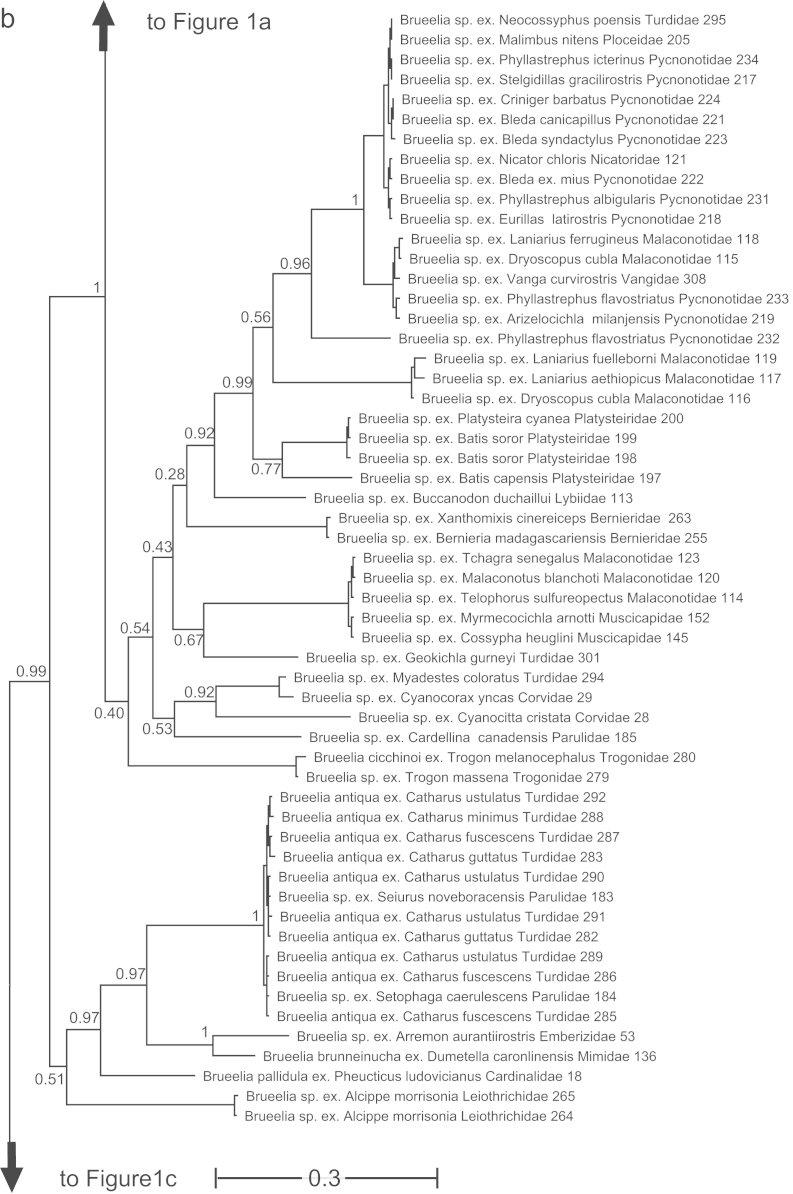

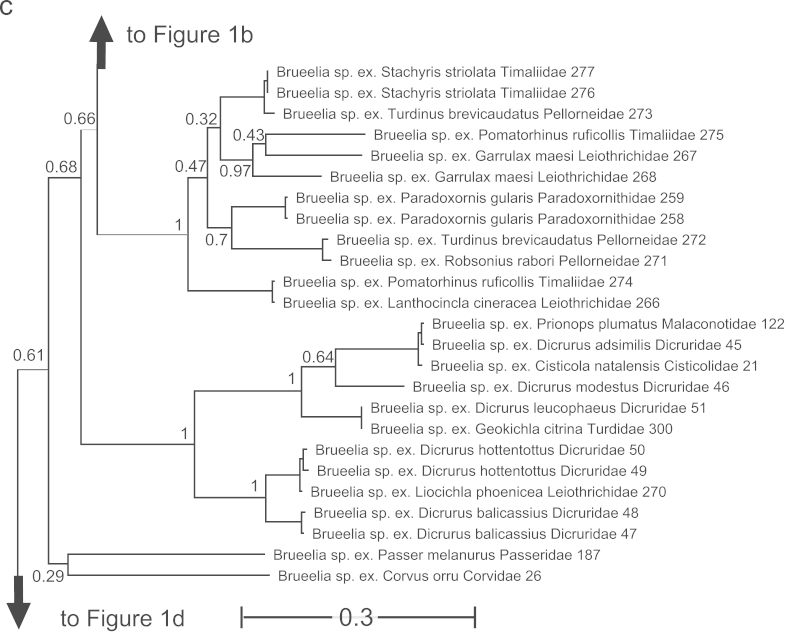

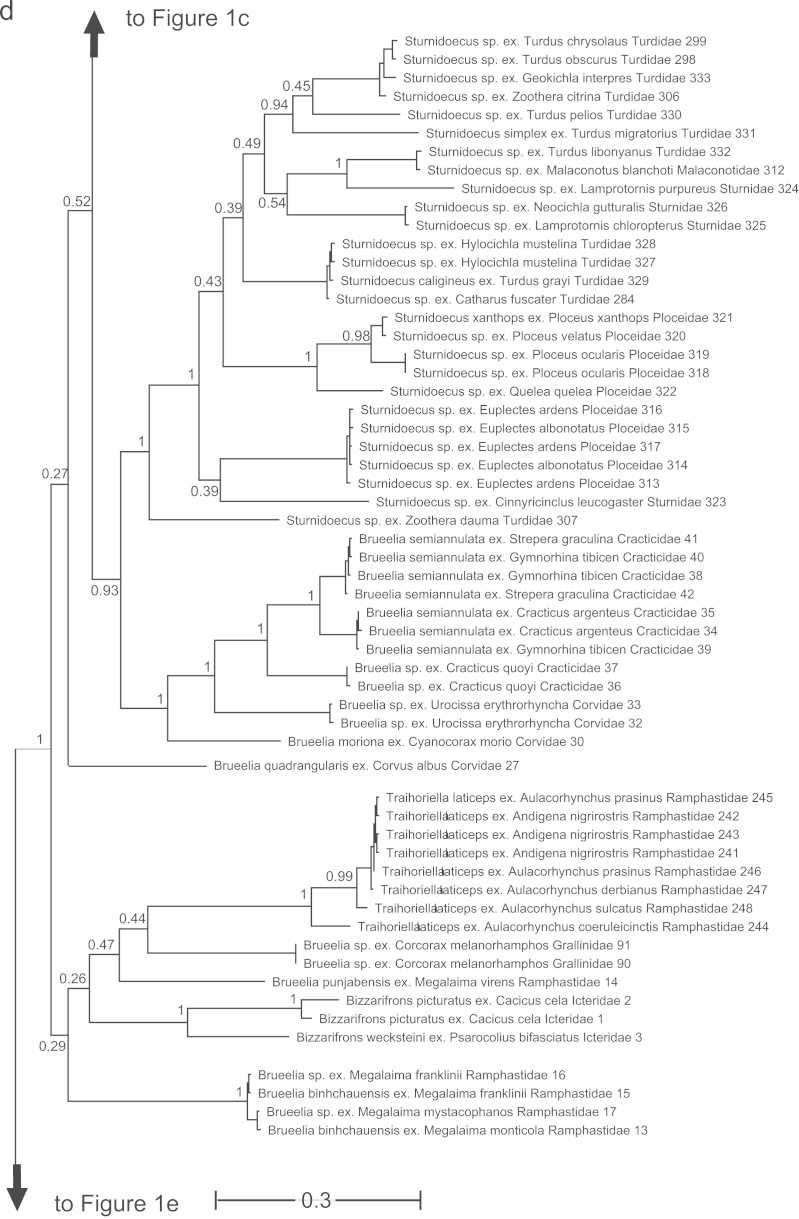

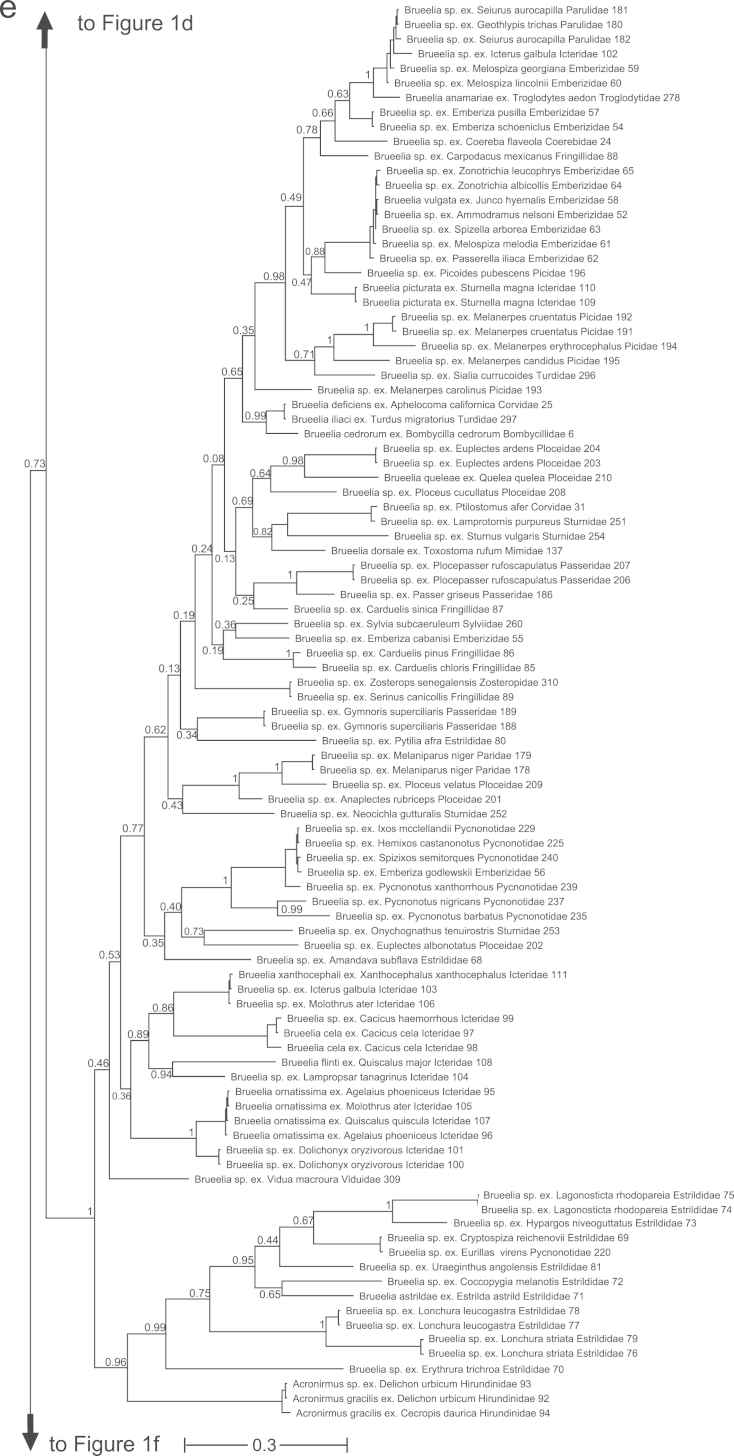

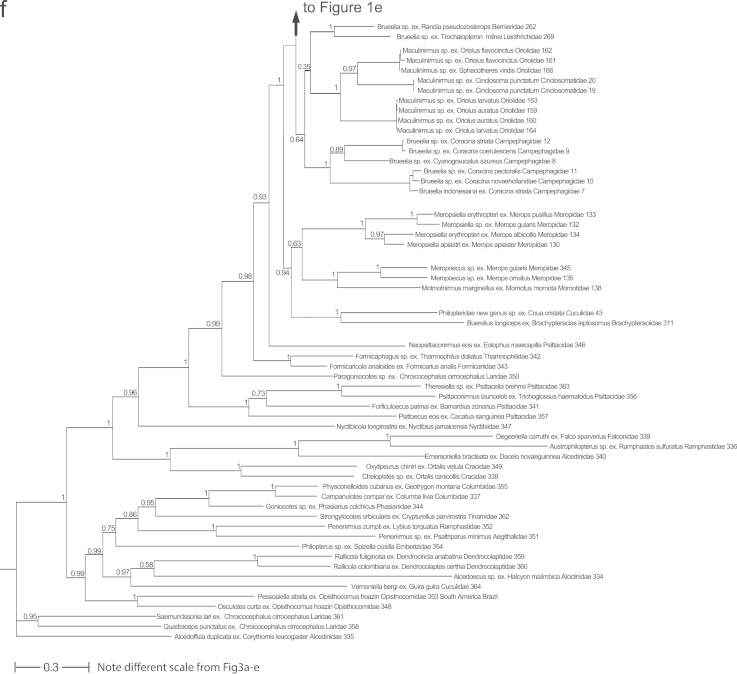
Consensus tree from Bayesian analysis of combined COI and EF-1α sequences for *Brueelia*-complex species and outgroup taxa. Branches proportional to substitutions per site for the consensus tree (scale indicated). Numbers associated with nodes are posterior probabilities for the clade from a 10 million generation MCMC analysis, sampled every 1000 generations and excluding the first 1 million generations as burn-in (values<0.5, and values associated with short terminal branches not shown here; all support values>0.5 are shown on Fig. 2). Numbers after taxonomic names refer to Supplemental Table 1. Louse taxonomy follows the classification of Price et al. [9] and subsequent publications. Host taxonomy follows Clements et al. [21] and Dickinson et al. [22]: host genus, species, and family are all indicated. Tree partitioned into six portions (a-f).

**Fig. 2:**
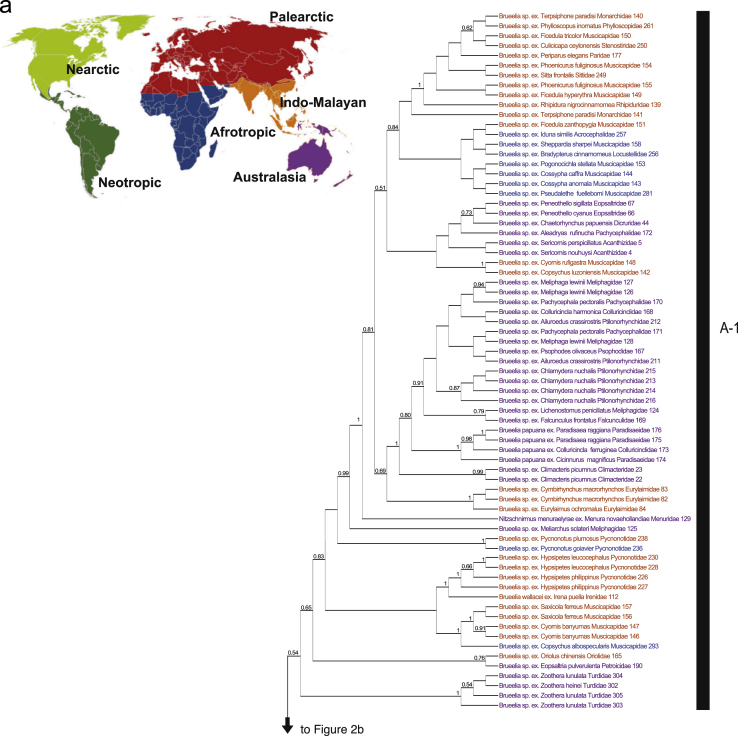

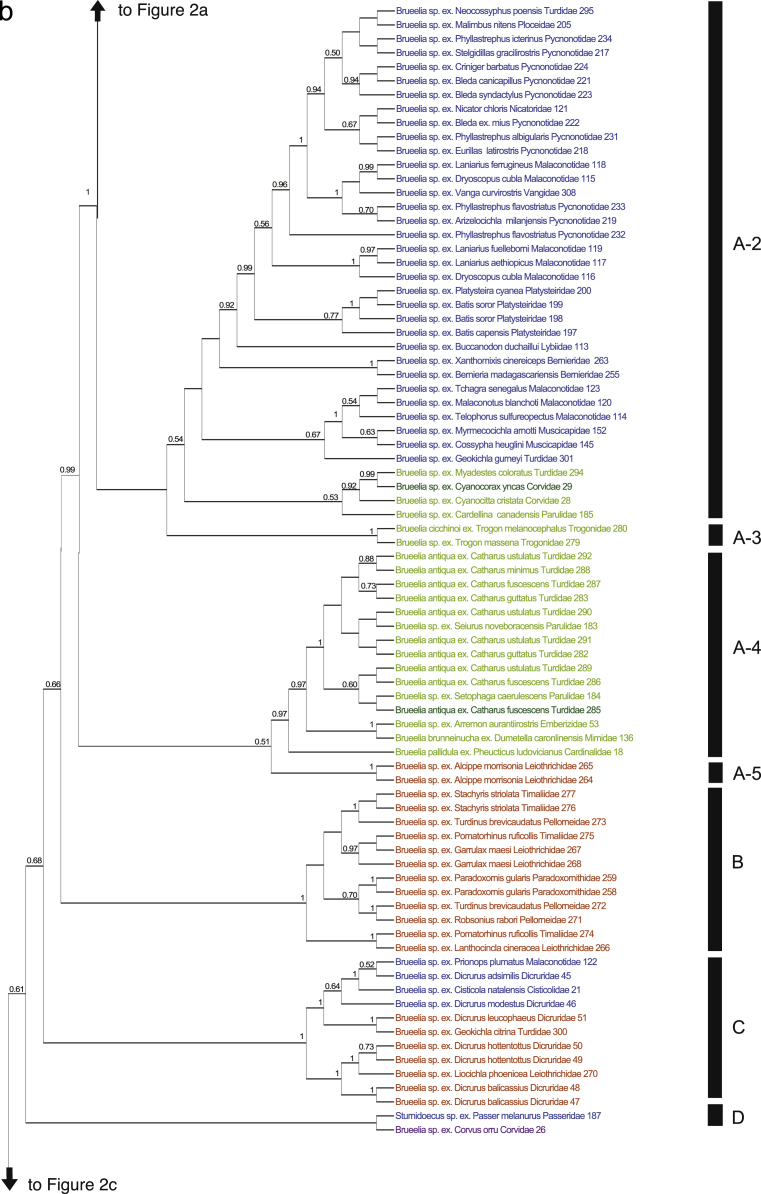

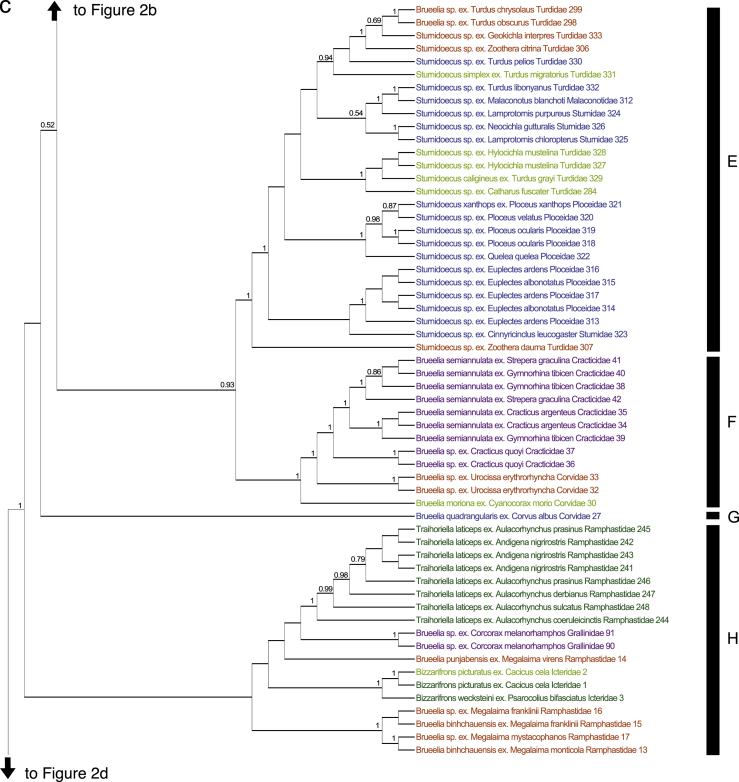

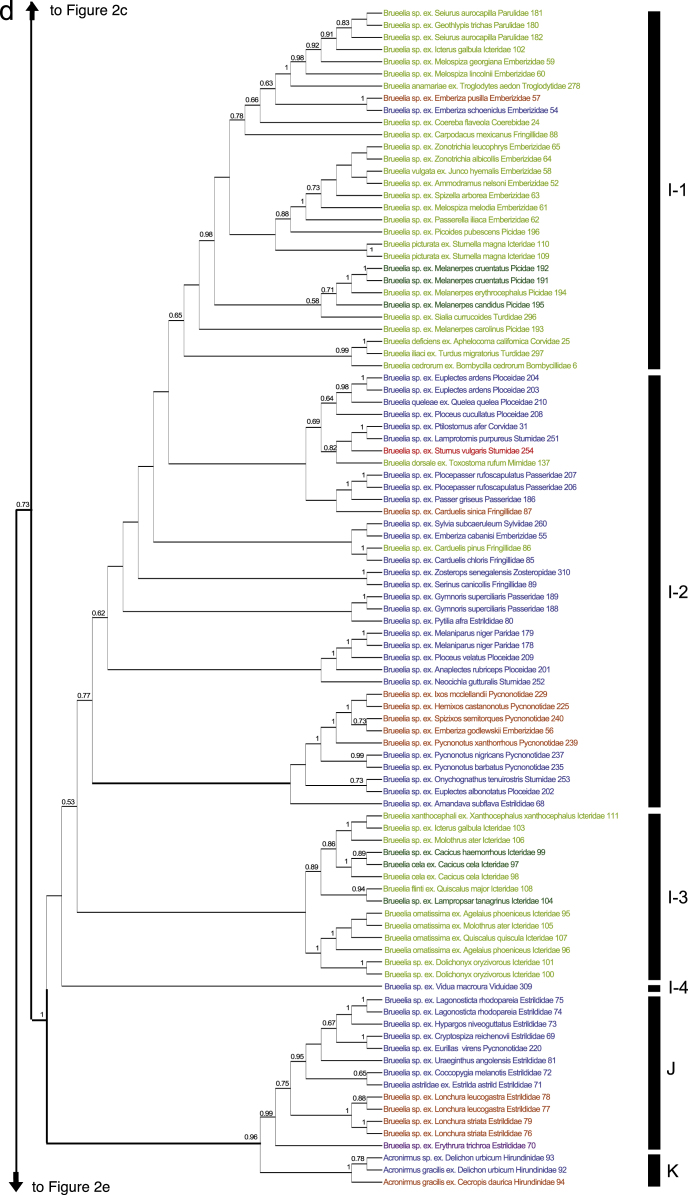

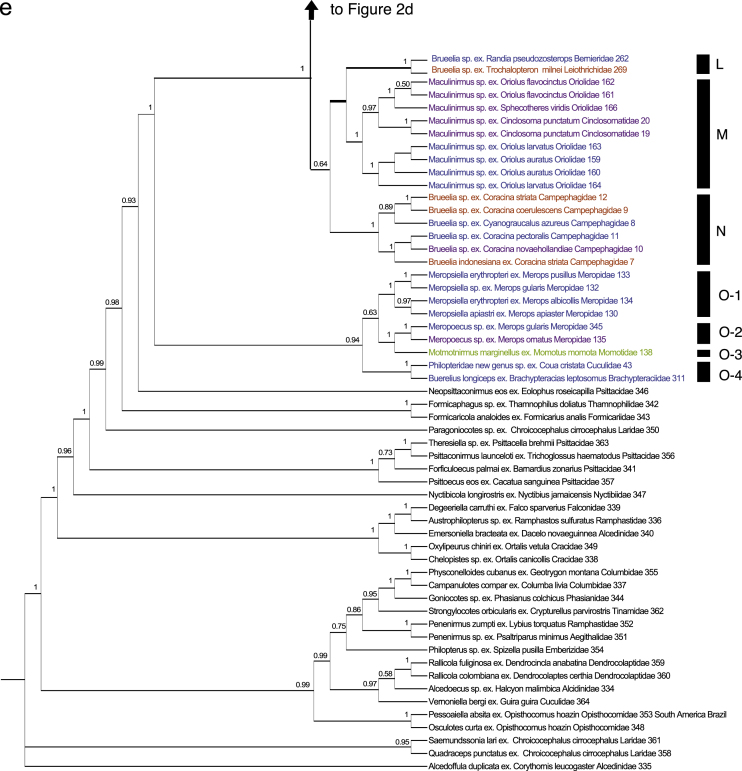
Cladogram of the consensus tree from a Bayesian analysis of combined COI and EF-1α sequences for *Brueelia*-complex species and outgroup taxa. Numbers associated with nodes are posterior probabilities calculated from 10 million MCMC generations sampled every 1000 and excluding the first 1 million generations as burn-in (values<0.5 not shown). Taxa colored to indicate geographic origin as indicated in map in 2a. Conventions as in Fig. 1. Tree partitioned into five portions (a–e).

A consensus tree from the Bayesian analysis of combined COI and EF-1a sequences for *Brueelia*-complex is shown in Fig. 1. A cladogram of the consensus tree from the Bayesian analysis is shown in Fig. 2.

## Appendix A Supplementary material

Supplementary material: Table 1 (pdf format)

Supplementary material: Table 1 (Excel Format)
