# Supplementary material for: Data supporting a molecular phylogeny of the hyper-diverse genus Brueelia
Source: Data Brief. 2015 Nov 2;5:1078–91. doi: 10.1016/j.dib.2015.10.022 (PMC4688976; doi:10.1016/j.dib.2015.10.022)
Supplement: Supplementary file 1 — Supplementary material: Table 1 (pdf format) [file mmc1.pdf]

Bush et al.:Data supporting a molecular phylogeny of the hyper-diverse genus *Brueelia*

Table 1: List of studied specimens, their voucher numbers, host associations, geographic origin, and genbank accession numbers.

*Institutions where slide-mounted voucher specimens of lice are deposited: FMNH - Field Museum of Natural History, Chicago, IL; INHS - Illinois Natural History Survey, Champaign-Urbana, IL; PIPeR - Price Institutue of Parasite Research, University of Utah, Salt Lake City, UT; "–" indicates specimens for which there is not a voucher specimen.

												Genbank #	Genbank #
**Table#**	**Louse voucher**	**Deposition of lice***	**Louse Genus**	**Louse species**	**Host family**	**Host Genus**	**Host species**	**BioRegion**	**Country**	**5%OTU (n = 166 OTUs)**	**bGMYC OTU (n = 114 OTUs)**	**COI Accestion#**	**EF-1a Accession#**
1	Bipi.8.25.2011.1	FMNH	*Bizzarifrons*	*picturatus*	Icteridae	*Cacicus*	*cela*	Neotropic	Brazil	159	54	KT892065	KT892357
2	Bzsp.Cacel.8.31.2011.10	INHS	*Bizzarifrons*	*picturatus*	Icteridae	*Cacicus*	*cela*	Neotropic	Panama	114	54	KT892331	KT892621
3	Bisp.Psbi.8.25.2011.2	FMNH	*Bizzarifrons*	*wecksteini*	Icteridae	*Psarocolius*	*bifasciatus*	Neotropic	Brazil	155	1	KT892066	KT892358
4	Brsp.Senoh.9.6.2011.10	PIPeR	*Brueelia*	*sp.*	Acanthizidae	*Sericornis*	*nouhuysi*	Australasia	Guinea	113	114	KT892259	KT892549
5	Brsp.Seper.4.16.2012.1	PIPeR	*Brueelia*	*sp.*	Acanthizidae	*Sericornis*	*perspicillatus*	Australasia	Guinea	1	114	KT892260	KT892550
6	Brce.6.9.2011.5	FMNH	*Brueelia*	*cedrorum*	Bombycillidae	*Bombycilla*	*cedrorum*	Nearctic	USA	166	81	KT892073	KT892365
7	Brin.8.16.2011.7	FMNH	*Brueelia*	*indonesiana*	Campephagidae	*Coracina*	*striata*	Indo-Malayan	Phillipines	37	83	KT892079	KT892371
8	Brsp.Coazu.11.8.2011.6	INHS	*Brueelia*	*sp.*	Campephagidae	*Cyanograucalus*	*azureus*	Afrotropic	Ghana	140	14	KT892131	KT892423
9	Brsp.Cocoe.11.8.2011.3	PIPeR	*Brueelia*	*sp.*	Campephagidae	*Coracina*	*coerulescens*	Indo-Malayan	Philippines	39	62	KT892133	KT892425
10	Brsp.Conov.12.13.2011.7	PIPeR	*Brueelia*	*sp.*	Campephagidae	*Coracina*	*novaehollandiae*	Australasia	Australia	37	83	KT892140	KT892432
11	Brsp.Cope.11.29.2010.19	FMNH	*Brueelia*	*sp.*	Campephagidae	*Coracina*	*pectoralis*	Afrotropic	Malawi	136	83	KT892142	KT892434
12	Brsp.Costr.7.14.1999.10	PIPeR	*Brueelia*	*sp.*	Campephagidae	*Coracina*	*striata*	Indo-Malayan	Philippines	39	62	AY149390	AY149420
13	Brsp.Memon.10.5.1999.10	PIPeR	*Brueelia*	*binhchauensis*	Ramphastidae	*Megalaima*	*monticola*	Indo-Malayan	Malaysia	87	97	AY149388	AY149418
14	Brsp.Mevir.10.4.2011.4	PIPeR	*Brueelia*	*punjabensis*	Ramphastidae	*Megalaima*	*virens*	Indo-Malayan	China	83	24	KT892206	KT892497
15	BrspMefra420201218	PIPeR	*Brueelia*	*binhchauensis*	Ramphastidae	*Megalaima*	*franklinii*	Indo-Malayan	China	25	97	KT892306	KT892596
16	BrspMefra420201219	–	*Brueelia*	*sp.*	Ramphastidae	*Megalaima*	*franklinii*	Indo-Malayan	China	25	97	KT892307	KT892597
17	Pnsp.Memys.1.15.2000.15	PIPeR	*Brueelia*	*binhchauensis*	Ramphastidae	*Megalaima*	*mystacophanos*	Indo-Malayan	Malaysia	25	97	AY149389	AY149419
18	Brpa.8.29.2011.14	FMNH	*Brueelia*	*pallidula*	Cardinalidae	*Pheucticus*	*ludovicianus*	Nearctic	USA	141	10	KT892089	KT892381
19	Brsp.Cipun.12.16.2011.4	PIPeR	*Maculinirmus*	*sp.*	Cinclosomatidae	*Cinclosoma*	*punctatum*	Australasia	Guinea	31	61	KT892125	KT892417
20	Brsp.Cipun.8.31.2011.1	–	*Maculinirmus*	*sp.*	Cinclosomatidae	*Cinclosoma*	*punctatum*	Australasia	Australia	31	61	KT892126	KT892418
21	Brsp.Cina.2.9.2011.26	FMNH	*Brueelia*	*sp.*	Cisticolidae	*Cisticola*	*natalensis*	Afrotropic	Malawi	21	104	KT892124	KT892416
22	Brsp.Clpic.05.21.2012.05	PIPeR	*Brueelia*	*sp.*	Climacteridae	*Climacteris*	*picumnus*	Australasia	Australia	2	114	KT892127	KT892419
23	Brsp.Clpic.8.31.2011.3	PIPeR	*Brueelia*	*sp.*	Climacteridae	*Climacteris*	*picumnus*	Australasia	Australia	2	114	KT892128	KT892420
24	Brsp.Cofla.11.28.2011.12	INHS	*Brueelia*	*sp.*	Coerebidae	*Coereba*	*flaveola*	Neotropic	Panama	139	113	KT892134	KT892426
25	BrspApcal32620122	PIPeR	*Brueelia*	*deficiens*	Corvidae	*Aphelocoma*	*californica*	Nearctic	USA	55	81	KT892285	KT892575
26	Brsp.Coorr.05.30.2012.04	PIPeR	*Brueelia*	*sp.*	Corvidae	*Corvus*	*orru*	Australasia	Australia	137	15	KT892141	KT892433
27	BrspCoalb326201224B	PIPeR	*Brueelia*	*quadrangularis*	Corvidae	*Corvus*	*albus*	Afrotropic	Ghana	93	37	KT892287	KT892577
28	Brcl.8.16.2011.8	FMNH	*Brueelia*	*sp.*	Corvidae	*Cyanocitta*	*cristata*	Nearctic	USA	123	5	KT892074	KT892366
29	Brho.5.30.2012.15	FMNH	*Brueelia*	*sp.*	Corvidae	*Cyanocorax*	*yncas*	Neotropic	Venezuela	49	57	KT892077	KT892369
30	Brmor.4.7.1999.8	PIPeR	*Brueelia*	*moriona*	Corvidae	*Cyanocorax*	*morio*	Neotropic	Mexico	134	8	AY149400	AY149430
31	Brsp.Ptafe.4.16.2012.11	INHS	*Brueelia*	*sp.*	Corvidae	*Ptilostomus*	*afer*	Afrotropic	Ghana	44	67	KT892247	KT892537
32	Brsp.Urery.12.13.2011.3	PIPeR	*Brueelia*	*sp.*	Corvidae	*Urocissa*	*erythrorhyncha*	Indo-Malayan	China	102	74	KT892272	KT892562
33	BrspUeery326201216A	PIPeR	*Brueelia*	*sp.*	Corvidae	*Urocissa*	*erythrorhyncha*	Indo-Malayan	China	108	74	KT892324	KT892614
34	Brsp.Crarg.05.30.2012.05	PIPeR	*Brueelia*	*semiannulata*	Cracticidae	*Cracticus*	*argenteus*	Australasia	Australia	20	109	KT892143	KT892435
35	Brsp.Crarg.12.13.2011.8	PIPeR	*Brueelia*	*semiannulata*	Cracticidae	*Cracticus*	*argenteus*	Australasia	Australia	20	109	KT892144	KT892436
36	Brsp.Crquo.05.30.2012.01	PIPeR	*Brueelia*	*sp.*	Cracticidae	*Cracticus*	*quoyi*	Australasia	Australia	40	64	KT892146	KT892438

Table#	Louse voucher	Deposition of lice*	Louse Genus	Louse species	Host family	Host Genus	Host species	BioRegion	Country	5%OTU (n = 166 OTUs)	bGMYC OTU (n = 114 OTUs)	COI Accestion#	EF-1a Accession#
37	Brsp.Crquo.05.30.2012.02	PIPeR	*Brueelia*	*sp.*	Cracticidae	*Cracticus*	*quoyi*	Australasia	Australia	40	64	KT892147	KT892439
38	Brsp.Gytib.05.30.2012.12	PIPeR	*Brueelia*	*semiannulata*	Cracticidae	*Gymnorhina*	*tibicen*	Australasia	Australia	14	109	KT892173	KT892465
39	Brsp.Gytib.12.13.2011.10	PIPeR	*Brueelia*	*semiannulata*	Cracticidae	*Gymnorhina*	*tibicen*	Australasia	Australia	20	109	KT892174	KT892466
40	Brsp.Gytib.9.6.2011.6	–	*Brueelia*	*semiannulata*	Cracticidae	*Gymnorhina*	*tibicen*	Australasia	Australia	14	109	KT892175	KT892467
41	Brsp.Stgra.05.30.2012.09	PIPeR	*Brueelia*	*semiannulata*	Cracticidae	*Strepera*	*graculina*	Australasia	Australia	14	109	KT892264	KT892554
42	Brsp.Stgra.9.6.2011.4	PIPeR	*Brueelia*	*semiannulata*	Cracticidae	*Strepera*	*graculina*	Australasia	Australia	14	109	KT892265	KT892555
43	Cusp.Cocr.8.25.2009.9	FMNH	Philopteridae	*sp.*	Cuculidae	*Coua*	*cristata*	Afrotropic	Madagascar	115	46	KT892332	KT892622
44	Brsp.Chpap.11.28.2011.6	PIPeR	*Brueelia*	*sp.*	Dicruridae	*Chaetorhynchus*	*papuensis*	Australasia	Guinea	1	114	KT892122	KT892414
45	Brsp.Diad.1.25.2011.4	FMNH	*Brueelia*	*sp.*	Dicruridae	*Dicrurus*	*adsimilis*	Afrotropic	Malawi	21	104	KT892153	KT892445
46	Brsp.Dimod.4.16.2012.6	INHS	*Brueelia*	*sp.*	Dicruridae	*Dicrurus*	*modestus*	Afrotropic	Ghana	133	104	KT892154	KT892446
47	BrspDibal42820122	PIPeR	*Brueelia*	*sp.*	Dicruridae	*Dicrurus*	*balicassius*	Indo-Malayan	Philippines	34	105	KT892291	KT892581
48	BrspDibal42820123	PIPeR	*Brueelia*	*sp.*	Dicruridae	*Dicrurus*	*balicassius*	Indo-Malayan	Philippines	34	105	KT892292	KT892582
49	BrspDihot420201210	PIPeR	*Brueelia*	*sp.*	Dicruridae	*Dicrurus*	*hottentottus*	Indo-Malayan	China	56	105	KT892293	KT892583
50	BrspDihot42020129	PIPeR	*Brueelia*	*sp.*	Dicruridae	*Dicrurus*	*hottentottus*	Indo-Malayan	China	91	105	KT892294	KT892584
51	BrspDileu415201231	PIPeR	*Brueelia*	*sp.*	Dicruridae	*Dicrurus*	*leucophaeus*	Indo-Malayan	China	36	104	KT892295	KT892585
52	Brsp.Amne.8.16.2011.3	FMNH	*Brueelia*	*sp.*	Emberizidae	*Ammodramus*	*nelsoni*	Nearctic	USA	5	113	KT892096	KT892388
53	Brsp.Araur.8.31.2011.12	INHS	*Brueelia*	*sp.*	Emberizidae	*Arremon*	*aurantiirostris*	Neotropic	Panama	146	55	KT892102	KT892394
54	Brbla.Emsch.1.23.2012.4	PIPeR	*Brueelia*	*sp.*	Emberizidae	*Emberiza*	*schoeniclus*	Palearctic	Sweden	162	113	KT892071	KT892363
55	Brsp.Emca.1.25.2011.8	FMNH	*Brueelia*	*sp.*	Emberizidae	*Emberiza*	*cabanisi*	Afrotropic	Malawi	130	16	KT892157	KT892449
56	Brsp.Emgod.12.11.2011.1	PIPeR	*Brueelia*	*sp.*	Emberizidae	*Emberiza*	*godlewskii*	Indo-Malayan	China	10	102	KT892158	KT892450
57	Brsp.Empus.10.4.2011.13	PIPeR	*Brueelia*	*sp.*	Emberizidae	*Emberiza*	*pusilla*	Indo-Malayan	China	129	113	KT892159	KT892451
58	Brvu.6.27.2006.28	FMNH	*Brueelia*	*vulgata*	Emberizidae	*Junco*	*hyemalis*	Nearctic	USA	5	113	FJ171236	FJ171260
59	Brsp.Mege.6.27.2006.17	FMNH	*Brueelia*	*sp.*	Emberizidae	*Melospiza*	*georgiana*	Nearctic	USA	7	113	FJ171232	FJ171256
60	Brsp.Meli.8.29.2011.7	FMNH	*Brueelia*	*sp.*	Emberizidae	*Melospiza*	*lincolnii*	Nearctic	USA	7	113	KT892201	KT892492
61	Brsp.Meme.8.29.2011.8	FMNH	*Brueelia*	*sp.*	Emberizidae	*Melospiza*	*melodia*	Nearctic	USA	5	113	KT892202	KT892493
62	Brsp.Pail.5.30.2012.17	FMNH	*Brueelia*	*sp.*	Emberizidae	*Passerella*	*iliaca*	Nearctic	USA	5	113	KT892223	KT892513
63	Brsp.Spar.9.14.2011.3	FMNH	*Brueelia*	*sp.*	Emberizidae	*Spizella*	*arborea*	Nearctic	USA	5	113	KT892261	KT892551
64	Brsp.Zoal.6.27.2006.19	FMNH	*Brueelia*	*sp.*	Emberizidae	*Zonotrichia*	*albicollis*	Nearctic	USA	5	113	FJ171234	FJ171258
65	Brsp.Zole.6.27.2006.21	FMNH	*Brueelia*	*sp.*	Emberizidae	*Zonotrichia*	*leucophrys*	Nearctic	USA	5	113	FJ171235	FJ171259
66	Brsp.Pacya.4.16.2012.2	PIPeR	*Brueelia*	*sp.*	Eopsaltridae	*Peneothello*	*cyanus*	Australasia	Guinea	1	114	KT892221	KT892511
67	Brsp.Pesig.11.28.2011.3	PIPeR	*Brueelia*	*sp.*	Eopsaltridae	*Peneothello*	*sigillata*	Australasia	Guinea	1	114	KT892231	KT892521
68	Brsp.Amsu.2.9.2011.40	FMNH	*Brueelia*	*sp.*	Estrildidae	*Amandava*	*subflava*	Afrotropic	Malawi	143	11	KT892097	KT892389
69	Brsp.Crre.11.29.2010.16	FMNH	*Brueelia*	*sp.*	Estrildidae	*Cryptospiza*	*reichenovii*	Afrotropic	Malawi	41	59	KT892148	KT892440
70	Brsp.Ertri.11.28.2011.2	PIPeR	*Brueelia*	*sp.*	Estrildidae	*Erythrura*	*trichroa*	Australasia	Guinea	126	18	KT892162	KT892454
71	Bras.11.15.2010.14	FMNH	*Brueelia*	*astrildae*	Estrildidae	*Estrilda*	*astrild*	Afrotropic	Malawi	157	4	KT892070	KT892362
72	Brsp.Esme.11.15.2010.13	FMNH	*Brueelia*	*sp.*	Estrildidae	*Coccopygia*	*melanotis*	Afrotropic	Malawi	125	19	KT892163	KT892455
73	Brsp.Hyni.11.29.2010.17	FMNH	*Brueelia*	*sp.*	Estrildidae	*Hypargos*	*niveoguttatus*	Afrotropic	Malawi	158	86	KT892178	KT892470
74	Brsp.Larh.5.30.2012.4	FMNH	*Brueelia*	*sp.*	Estrildidae	*Lagonosticta*	*rhodopareia*	Afrotropic	Mozambique	50	86	KT892187	KT892479
75	Brsp.Larh.5.30.2012.5	FMNH	*Brueelia*	*sp.*	Estrildidae	*Lagonosticta*	*rhodopareia*	Afrotropic	Mozambique	50	86	KT892188	KT892480
76	Brsp.Lostr.12.11.2011.7	PIPeR	*Brueelia*	*sp.*	Estrildidae	*Lonchura*	*striata*	Indo-Malayan	China	51	95	KT892191	KT892483
77	BrspLoleu42820125	–	*Brueelia*	*sp.*	Estrildidae	*Lonchura*	*leucogastra*	Indo-Malayan	Philippines	57	95	KT892302	KT892592

Table#	Louse voucher	Deposition of lice*	Louse Genus	Louse species	Host family	Host Genus	Host species	BioRegion	Country	5%OTU (n = 166 OTUs)	bGMYC OTU (n = 114 OTUs)	COI Accestion#	EF-1a Accession#
78	BrspLoleu42820126	PIPeR	*Brueelia*	*sp.*	Estrildidae	*Lonchura*	*leucogastra*	Indo-Malayan	Philippines	57	95	KT892303	KT892593
79	BrspLostr326201233	PIPeR	*Brueelia*	*sp.*	Estrildidae	*Lonchura*	*striata*	Indo-Malayan	China	51	95	KT892304	KT892594
80	Brsp.Pyaf.11.15.2010.16	FMNH	*Brueelia*	*sp.*	Estrildidae	*Pytilia*	*afra*	Afrotropic	Malawi	74	30	KT892248	KT892538
81	Brsp.Uran.2.9.2011.35	FMNH	*Brueelia*	*sp.*	Estrildidae	*Uraeginthus*	*angolensis*	Afrotropic	Malawi	103	33	KT892271	KT892561
82	Brsp.Cyma.10.15.2012.23	FMNH	*Brueelia*	*sp.*	Eurylaimidae	*Cymbirhynchus*	*macrorhynchos*	Indo-Malayan	Malaysia	24	114	KT892150	KT892442
83	Brsp.Cyma.8.16.2011.10	PIPeR	*Brueelia*	*sp.*	Eurylaimidae	*Cymbirhynchus*	*macrorhynchos*	Indo-Malayan	Malaysia	24	114	KT892151	KT892443
84	Brsp.Euoc.10.15.2012.22	–	*Brueelia*	*sp.*	Eurylaimidae	*Eurylaimus*	*ochromalus*	Indo-Malayan	Malaysia	24	114	KT892167	KT892459
85	Brbre.Cachl.4.16.2012.9	PIPeR	*Brueelia*	*sp.*	Fringillidae	*Carduelis*	*chloris*	Palearctic	UK	165	56	KT892072	KT892364
86	Brsp.Capi.8.16.2011.6	FMNH	*Brueelia*	*sp.*	Fringillidae	*Carduelis*	*pinus*	Nearctic	USA	145	56	KT892116	KT892408
87	Brsp.Casin.10.4.2011.11	PIPeR	*Brueelia*	*sp.*	Fringillidae	*Carduelis*	*sinica*	Indo-Malayan	China	144	13	KT892117	KT892409
88	Brsp.Camex.2.1.2000.8	FMNH	*Brueelia*	*sp.*	Fringillidae	*Carpodacus*	*mexicanus*	Nearctic	USA	147	113	AY149394	AY149424
89	Brsp.Seca.2.9.2011.29	FMNH	*Brueelia*	*sp.*	Fringillidae	*Serinus*	*canicollis*	Afrotropic	Malawi	42	73	KT892257	KT892547
90	Brsp.Comel.11.8.2011.1	PIPeR	*Brueelia*	*sp.*	Grallinidae	*Corcorax*	*melanorhamphos*	Australasia	Australia	35	63	KT892138	KT892430
91	Brsp.Comel.11.8.2011.2	PIPeR	*Brueelia*	*sp.*	Grallinidae	*Corcorax*	*melanorhamphos*	Australasia	Australia	35	63	KT892139	KT892431
92	Brgr.2.9.2011.37	FMNH	*Acronirmus*	*gracilis*	Hirundinidae	*Delichon*	*urbicum*	Afrotropic	Malawi	59	82	KT892075	KT892367
93	Brgra.Deurb.1.23.2012.3	PIPeR	*Acronirmus*	*sp.*	Hirundinidae	*Delichon*	*urbicum*	Palearctic	Sweden	59	82	KT892076	KT892368
94	Brsp.Hidau.10.25.2011.9	PIPeR	*Acronirmus*	*gracilis*	Hirundinidae	*Cecropis*	*daurica*	Indo-Malayan	Philippines	160	82	KT892177	KT892469
95	Brsp.Agph.8.16.2011.2	FMNH	*Brueelia*	*ornatissima*	Icteridae	*Agelaius*	*phoeniceus*	Nearctic	USA	13	108	KT892090	KT892382
96	BrspAgpho326201238B	PIPeR	*Brueelia*	*ornatissima*	Icteridae	*Agelaius*	*phoeniceus*	Nearctic	USA	13	108	KT892284	KT892574
97	Bram.8.16.2011.5	FMNH	*Brueelia*	*cela*	Icteridae	*Cacicus*	*cela*	Neotropic	Brazil	30	80	KT892068	KT892360
98	Brsp.Cacel.8.31.2011.11	INHS	*Brueelia*	*cela*	Icteridae	*Cacicus*	*cela*	Neotropic	Panama	30	80	KT892114	KT892406
99	Brsp.Cahae.10.12.1999.9	FMNH	*Brueelia*	*sp.*	Icteridae	*Cacicus*	*haemorrhous*	Neotropic	Brazil	30	80	AY149393	AY149423
100	Bror.8.16.2011.12	FMNH	*Brueelia*	*sp.*	Icteridae	*Dolichonyx*	*oryzivorous*	Nearctic	USA	64	108	KT892086	KT892378
101	Brsp.Door.6.13.2006.13	FMNH	*Brueelia*	*sp.*	Icteridae	*Dolichonyx*	*oryzivorous*	Nearctic	USA	64	108	FJ171230	FJ171254
102	Brsp.Icga.8.16.2011.14	FMNH	*Brueelia*	*sp.*	Icteridae	*Icterus*	*galbula*	Nearctic	USA	7	113	KT892182	KT892474
103	BrspIcgal326201240	–	*Brueelia*	*sp.*	Icteridae	*Icterus*	*galbula*	Nearctic	USA	26	88	KT892300	KT892590
104	Brsp.Lata.8.29.2011.1	FMNH	*Brueelia*	*sp.*	Icteridae	*Lampropsar*	*tanagrinus*	Neotropic	Brazil	150	21	KT892189	KT892481
105	Bror.8.29.2011.10	FMNH	*Brueelia*	*ornatissima*	Icteridae	*Molothrus*	*ater*	Nearctic	USA	13	108	KT892087	KT892379
106	Brsp.Moate.3.24.2001.3	INHS	*Brueelia*	*sp.*	Icteridae	*Molothrus*	*ater*	Nearctic	USA	26	88	KT892207	JX121686
107	Bror.8.29.2011.16	FMNH	*Brueelia*	*ornatissima*	Icteridae	*Quiscalus*	*quiscula*	Nearctic	USA	13	108	KT892088	KT892380
108	Brsp.Qumaj.11.8.2011.12	INHS	*Brueelia*	*flinti*	Icteridae	*Quiscalus*	*major*	Neotropic	Panama	117	31	KT892254	KT892544
109	BrspStmag326201246	PIPeR	*Brueelia*	*picturata*	Icteridae	*Sturnella*	*magna*	Nearctic	USA	106	76	KT892320	KT892610
110	BrspStmag326201247	PIPeR	*Brueelia*	*picturata*	Icteridae	*Sturnella*	*magna*	Nearctic	USA	107	76	KT892321	KT892611
111	BrspXaxan326201248	PIPeR	*Brueelia*	*xanthocephali*	Icteridae	*Xanthocephalus*	*xanthocephalus*	Nearctic	USA	26	88	KT892325	KT892615
112	Brsp.Irpu.6.27.2006.23	PIPeR	*Brueelia*	*wallacei*	Irenidae	*Irena*	*puella*	Indo-Malayan	Malaysia	152	103	FJ171231	FJ171255
113	Brsp.Budu.8.16.2011.4	FMNH	*Brueelia*	*sp.*	Lybiidae	*Buccanodon*	*duchaillui*	Afrotropic	Ghana	149	12	KT892113	KT892405
114	Brsp.Tesu.2.9.2011.33	FMNH	*Brueelia*	*sp.*	Malaconotidae	*Telophorus*	*sulfureopectus*	Afrotropic	Malawi	8	101	KT892269	KT892559
115	Brsp.Drcu.4.5.2011.14	FMNH	*Brueelia*	*sp.*	Malaconotidae	*Dryoscopus*	*cubla*	Afrotropic	Malawi	9	112	KT892155	KT892447
116	Brsp.Drcu.5.30.2012.1	FMNH	*Brueelia*	*sp.*	Malaconotidae	*Dryoscopus*	*cubla*	Afrotropic	Mozambique	29	84	KT892156	KT892448
117	Brsp.Laae.11.29.2010.18	FMNH	*Brueelia*	*sp.*	Malaconotidae	*Laniarius*	*aethiopicus*	Afrotropic	Malawi	29	84	KT892183	KT892475
118	Brsp.Lafe.11.29.2010.21	FMNH	*Brueelia*	*sp.*	Malaconotidae	*Laniarius*	*ferrugineus*	Afrotropic	Malawi	9	112	KT892184	KT892476

Table#	Louse voucher	Deposition of lice*	Louse Genus	Louse species	Host family	Host Genus	Host species	BioRegion	Country	5%OTU (n = 166 OTUs)	bGMYC OTU (n = 114 OTUs)	COI Accestion#	EF-1a Accession#
119	Brsp.Lafu.4.5.2011.16	FMNH	*Brueelia*	*sp.*	Malaconotidae	*Laniarius*	*fuelleborni*	Afrotropic	Malawi	29	84	KT892185	KT892477
120	Brsp.Mabl.1.25.2011.10	FMNH	*Brueelia*	*sp.*	Malaconotidae	*Malaconotus*	*blanchoti*	Afrotropic	Malawi	8	101	KT892192	KT892484
121	Brsp.Nichl.11.8.2011.8	INHS	*Brueelia*	*sp.*	Nicatoridae	*Nicator*	*chloris*	Afrotropic	Ghana	4	112	KT892213	KT892503
122	Brsp.Prpl.4.5.2011.18	FMNH	*Brueelia*	*sp.*	Malaconotidae	*Prionops*	*plumatus*	Afrotropic	Malawi	21	104	KT892245	KT892535
123	Brsp.Tcse.1.25.2011.14	FMNH	*Brueelia*	*sp.*	Malaconotidae	*Tchagra*	*senegalus*	Afrotropic	Malawi	8	101	KT892268	KT892558
124	Brsp.Lipen.05.21.2012.06	PIPeR	*Brueelia*	*sp.*	Meliphagidae	*Lichenostomus*	*penicillatus*	Australasia	Australia	2	114	KT892190	KT892482
125	Brsp.Mesc.5.30.2012.16	FMNH	*Brueelia*	*sp.*	Meliphagidae	*Meliarchus*	*sclateri*	Australasia	Islands	84	23	KT892205	KT892496
126	Brsp.Melew.05.21.2012.08	PIPeR	*Brueelia*	*sp.*	Meliphagidae	*Meliphaga*	*lewinii*	Australasia	Australia	2	114	KT892198	KT892489
127	Brsp.Melew.05.30.2012.10	PIPeR	*Brueelia*	*sp.*	Meliphagidae	*Meliphaga*	*lewinii*	Australasia	Australia	2	114	KT892199	KT892490
128	Brsp.Melew.9.6.2011.5	–	*Brueelia*	*sp.*	Meliphagidae	*Meliphaga*	*lewinii*	Australasia	Australia	2	114	KT892200	KT892491
129	Brsp.Menov.8.31.2011.5	PIPeR	*Nitzschnirmus*	*menuraelyrae*	Menuridae	*Menura*	*novaehollandiae*	Australasia	Australia	86	22	KT892203	KT892494
130	Brap.1.25.2011.1	FMNH	*Meropsiella*	*apiastri*	Meropidae	*Merops*	*apiaster*	Afrotropic	Malawi	154	3	KT892069	KT892361
131	–	–	–	–	–	–	–	–	–	–	–		
132	Brsp.Megul.4.17.2000.2	INHS	*Meropsiella*	*sp.*	Meropidae	*Merops*	*gularis*	Afrotropic	Ghana	88	68	KT892197	AY314827
133	Brsp.Mepu.4.5.2011.8	PIPeR	*Meropsiella*	*erythropteri*	Meropidae	*Merops*	*pusillus*	Afrotropic	Malawi	85	68	KT892204	KT892495
134	BrspMealb326201225A	INHS	*Meropsiella*	*erythropteri*	Meropidae	*Merops*	*albicollis*	Afrotropic	Ghana	94	39	KT892305	KT892595
135	BrspMeorn326201220	PIPeR	*Meropoecus*	*sp.*	Meropidae	*Merops*	*ornatus*	Australasia	Australia	95	40	KT892308	KT892598
136	Brbr.6.27.2006.20	FMNH	*Brueelia*	*brunneinucha*	Mimidae	*Dumetella*	*caronlinensis*	Nearctic	USA	164	55	FJ171223	FJ171246
137	Brdo.6.27.2006.24	FMNH	*Brueelia*	*dorsale*	Mimidae	*Toxostoma*	*rufum*	Nearctic	USA	127	6	FJ171224	FJ171247
138	Brmar.1.27.1999.11	PIPeR	*Motmotnirmus*	*marginellus*	Momotidae	*Momotus*	*momota*	Neotropic	Mexico	132	7	AY149401	AY149431
139	Brsp.Rhnig.7.14.1999.11	PIPeR	*Brueelia*	*sp.*	Rhipiduridae	*Rhipidura*	*nigrocinnamomea*	Indo-Malayan	Philippines	22	114	AY149384	AY149414
140	BrspTepar41520126	PIPeR	*Brueelia*	*sp.*	Monarchidae	*Terpsiphone*	*paradisi*	Indo-Malayan	China	1	114	KT892322	KT892612
141	BrspTepar41520127	PIPeR	*Brueelia*	*sp.*	Monarchidae	*Terpsiphone*	*paradisi*	Indo-Malayan	China	22	114	KT892323	KT892613
142	Brsp.Coluz.10.25.2011.16	PIPeR	*Brueelia*	*sp.*	Muscicapidae	*Copsychus*	*luzoniensis*	Indo-Malayan	Philippines	32	114	KT892137	KT892429
143	Brsp.Coan.1.25.2011.2	FMNH	*Brueelia*	*sp.*	Muscicapidae	*Cossypha*	*anomala*	Afrotropic	Malawi	1	114	KT892130	KT892422
144	Brsp.Coca.1.25.2011.7	FMNH	*Brueelia*	*sp.*	Muscicapidae	*Cossypha*	*caffra*	Afrotropic	Malawi	1	114	KT892132	KT892424
145	Brsp.Cohe.4.5.2011.7	FMNH	*Brueelia*	*sp.*	Muscicapidae	*Cossypha*	*heuglini*	Afrotropic	Malawi	8	101	KT892136	KT892428
146	BrspCyban415201219	PIPeR	*Brueelia*	*sp.*	Muscicapidae	*Cyornis*	*banyumas*	Indo-Malayan	China	19	100	KT892288	KT892578
147	BrspCyban415201220	PIPeR	*Brueelia*	*sp.*	Muscicapidae	*Cyornis*	*banyumas*	Indo-Malayan	China	19	100	KT892289	KT892579
148	BrspCyruf42820121	PIPeR	*Brueelia*	*sp.*	Muscicapidae	*Cyornis*	*rufigastra*	Indo-Malayan	Philippines	32	114	KT892290	KT892580
149	Brsp.Fihyp.7.14.1999.2	PIPeR	*Brueelia*	*sp.*	Muscicapidae	*Ficedula*	*hyperythra*	Indo-Malayan	Philippines	1	114	AY149410	AY149411
150	Brsp.Fitri.10.4.2011.14	PIPeR	*Brueelia*	*sp.*	Muscicapidae	*Ficedula*	*tricolor*	Indo-Malayan	China	122	114	KT892169	KT892461
151	BrspFizan415201225	PIPeR	*Brueelia*	*sp.*	Muscicapidae	*Ficedula*	*zanthopygia*	Indo-Malayan	China	89	114	KT892296	KT892586
152	Brsp.Myar.4.5.2011.17	FMNH	*Brueelia*	*sp.*	Muscicapidae	*Myrmecocichla*	*arnotti*	Afrotropic	Malawi	8	101	KT892208	KT892498
153	Brsp.Post.2.9.2011.34	FMNH	*Brueelia*	*sp.*	Muscicapidae	*Pogonocichla*	*stellata*	Afrotropic	Malawi	1	114	KT892244	KT892534
154	BrspRhful41520124	PIPeR	*Brueelia*	*sp.*	Muscicapidae	*Phoenicurus*	*fuliginosus*	Indo-Malayan	China	1	114	KT892315	KT892605
155	BrspRhful41520125	PIPeR	*Brueelia*	*sp.*	Muscicapidae	*Phoenicurus*	*fuliginosus*	Indo-Malayan	China	22	114	KT892316	KT892606
156	BrspSafer420201215	PIPeR	*Brueelia*	*sp.*	Muscicapidae	*Saxicola*	*ferreus*	Indo-Malayan	China	19	100	KT892317	KT892607
157	BrspSafer420201216	PIPeR	*Brueelia*	*sp.*	Muscicapidae	*Saxicola*	*ferreus*	Indo-Malayan	China	19	100	KT892318	KT892608
158	Brsp.Ersh.1.25.2011.16	FMNH	*Brueelia*	*sp.*	Muscicapidae	*Sheppardia*	*sharpei*	Afrotropic	Malawi	1	114	KT892161	KT892453
159	Brsp.Orau.11.29.2010.15	FMNH	*Maculinirmus*	*sp.*	Oriolidae	*Oriolus*	*auratus*	Afrotropic	Malawi	18	98	KT892215	KT892505

Table#	Louse voucher	Deposition of lice*	Louse Genus	Louse species	Host family	Host Genus	Host species	BioRegion	Country	5%OTU (n = 166 OTUs)	bGMYC OTU (n = 114 OTUs)	COI Accestion#	EF-1a Accession#
160	Brsp.Orau.4.5.2011.3	FMNH	*Maculinirmus*	*sp.*	Oriolidae	*Oriolus*	*auratus*	Afrotropic	Malawi	18	98	KT892216	KT892506
161	Brsp.Orfla.05.30.2012.03	PIPeR	*Maculinirmus*	*sp.*	Oriolidae	*Oriolus*	*flavocinctus*	Australasia	Australia	27	89	KT892217	KT892507
162	Brsp.Orfla.12.13.2011.11	PIPeR	*Maculinirmus*	*sp.*	Oriolidae	*Oriolus*	*flavocinctus*	Australasia	Australia	27	89	KT892218	KT892508
163	Brsp.Orla.5.30.2012.18	FMNH	*Maculinirmus*	*sp.*	Oriolidae	*Oriolus*	*larvatus*	Afrotropic	Mozambique	18	98	KT892219	KT892509
164	Brsp.Orla.5.30.2012.3	FMNH	*Maculinirmus*	*sp.*	Oriolidae	*Oriolus*	*larvatus*	Afrotropic	Mozambique	18	98	KT892220	KT892510
165	BrspOrchi420201223	PIPeR	*Brueelia*	*sp.*	Oriolidae	*Oriolus*	*chinensis*	Indo-Malayan	China	99	41	KT892311	KT892601
166	Brsp.Spvir.12.13.2011.12	PIPeR	*Maculinirmus*	*sp.*	Oriolidae	*Sphecotheres*	*viridis*	Australasia	Australia	27	89	KT892263	KT892553
167	Brsp.Psoli.05.30.2012.11	PIPeR	*Brueelia*	*sp.*	Psophodidae	*Psophodes*	*olivaceus*	Australasia	Australia	2	114	KT892246	KT892536
168	Brsp.Cohar.8.31.2011.4	PIPeR	*Brueelia*	*sp.*	Colluricinclidae	*Colluricincla*	*harmonica*	Australasia	Australia	2	114	KT892135	KT892427
169	Brsp.Fafro.05.21.2012.01	PIPeR	*Brueelia*	*sp.*	Falcunculidae	*Falcunculus*	*frontatus*	Australasia	Australia	2	114	KT892168	KT892460
170	Brsp.Papec.05.21.2012.07	PIPeR	*Brueelia*	*sp.*	Pachycephalidae	*Pachycephala*	*pectoralis*	Australasia	Australia	2	114	KT892226	KT892516
171	Brsp.Papec.9.6.2011.3	PIPeR	*Brueelia*	*sp.*	Pachycephalidae	*Pachycephala*	*pectoralis*	Australasia	Australia	2	114	KT892227	KT892517
172	Brsp.Paruf.4.16.2012.3	PIPeR	*Brueelia*	*sp.*	Pachycephalidae	*Aleadryas*	*rufinucha*	Australasia	Guinea	1	114	KT892230	KT892520
173	Brsp.Pifer.11.28.2011.7	PIPeR	*Brueelia*	*papuana*	Colluricinclidae	*Colluricincla*	*ferruginea*	Australasia	Guinea	15	114	KT892238	KT892528
174	BrspCimag121620117	PIPeR	*Brueelia*	*papuana*	Paradisaeidae	*Cicinnurus*	*magnificus*	Australasia	Guinea	15	114	KT892286	KT892576
175	Brsp.Parag.11.28.2011.5	PIPeR	*Brueelia*	*papuana*	Paradisaeidae	*Paradisaea*	*raggiana*	Australasia	Guinea	15	114	KT892228	KT892518
176	Brsp.Parag.12.16.2011.2	PIPeR	*Brueelia*	*papuana*	Paradisaeidae	*Paradisaea*	*raggiana*	Australasia	Guinea	15	114	KT892229	KT892519
177	Brsp.Paele.7.14.1999.3	PIPeR	*Brueelia*	*sp.*	Paridae	*Periparus*	*elegans*	Indo-Malayan	Philippines	1	114	AY149382	AY149412
178	Brsp.Pani.2.9.2011.32	FMNH	*Brueelia*	*sp.*	Paridae	*Melaniparus*	*niger*	Afrotropic	Malawi	48	96	KT892225	KT892515
179	Brsp.Panig.1.12.1999.11	–	*Brueelia*	*sp.*	Paridae	*Melaniparus*	*niger*	Afrotropic	South Africa	48	96	AY149391	AY149421
180	Brsp.Getr.8.16.2011.13	FMNH	*Brueelia*	*sp.*	Parulidae	*Geothlypis*	*trichas*	Nearctic	USA	7	113	KT892172	KT892464
181	Brsp.Seau.6.27.2006.22	FMNH	*Brueelia*	*sp.*	Parulidae	*Seiurus*	*aurocapilla*	Nearctic	USA	7	113	FJ171233	FJ171257
182	Brsp.Seau.9.14.2011.1	FMNH	*Brueelia*	*sp.*	Parulidae	*Seiurus*	*aurocapilla*	Nearctic	USA	7	113	KT892256	KT892546
183	Brsp.Seno.9.14.2011.2	FMNH	*Brueelia*	*sp.*	Parulidae	*Seiurus*	*noveboracensis*	Nearctic	USA	3	111	KT892258	KT892548
184	Brsp.Deca.8.16.2011.11	FMNH	*Brueelia*	*sp.*	Parulidae	*Setophaga*	*caerulescens*	Nearctic	USA	3	111	KT892152	KT892444
185	Brsp.Wica.9.14.2011.8	FMNH	*Brueelia*	*sp.*	Parulidae	*Cardellina*	*canadensis*	Nearctic	USA	100	35	KT892275	KT892565
186	Brsp.Pagr.11.15.2010.10	FMNH	*Brueelia*	*sp.*	Passeridae	*Passer*	*griseus*	Afrotropic	Malawi	77	90	KT892222	KT892512
187	Brsp.Pame.8.29.2011.12	PIPeR	*Brueelia*	*sp.*	Passeridae	*Passer*	*melanurus*	Afrotropic	South Africa	76	27	KT892224	KT892514
188	Brsp.Pesu.1.25.2011.11	FMNH	*Brueelia*	*sp.*	Passeridae	*Gymnoris*	*superciliaris*	Afrotropic	Malawi	47	70	KT892232	KT892522
189	Brsp.Pesu.2.9.2011.38	FMNH	*Brueelia*	*sp.*	Passeridae	*Gymnoris*	*superciliaris*	Afrotropic	Malawi	47	70	KT892233	KT892523
190	Brsp.Eopul.12.13.2011.9	PIPeR	*Brueelia*	*sp.*	Petroicidae	*Eopsaltria*	*pulverulenta*	Australasia	Australia	128	17	KT892160	KT892452
191	Brsp.Mecr.8.29.2011.3	FMNH	*Brueelia*	*sp.*	Picidae	*Melanerpes*	*cruentatus*	Neotropic	Brazil	52	87	KT892195	KT892487
192	Brsp.Mecru.11.28.2011.1	FMNH	*Brueelia*	*sp.*	Picidae	*Melanerpes*	*cruentatus*	Neotropic	Peru	52	87	KT892196	KT892488
193	Brst.8.29.2011.2	FMNH	*Brueelia*	*sp.*	Picidae	*Melanerpes*	*carolinus*	Nearctic	USA	111	45	KT892329	KT892619
194	Brst.8.29.2011.4	FMNH	*Brueelia*	*sp.*	Picidae	*Melanerpes*	*erythrocephalus*	Nearctic	USA	112	87	KT892330	KT892620
195	Pisp.Mecan.1.15.2000.12	FMNH	*Brueelia*	*sp.*	Picidae	*Melanerpes*	*candidus*	Neotropic	Bolivia	72	49	AY149395	AY149425
196	Brst.8.29.2011.15	FMNH	*Brueelia*	*sp.*	Picidae	*Picoides*	*pubescens*	Nearctic	USA	110	44	KT892328	KT892618
197	Brsp.Badi.1.25.2011.13	FMNH	*Brueelia*	*sp.*	Platysteiridae	*Batis*	*capensis*	Afrotropic	Malawi	148	93	KT892105	KT892397
198	Brsp.Baso.5.30.2012.6	FMNH	*Brueelia*	*sp.*	Platysteiridae	*Batis*	*soror*	Afrotropic	Mozambique	23	93	KT892106	KT892398
199	Brsp.Baso.5.30.2012.7	FMNH	*Brueelia*	*sp.*	Platysteiridae	*Batis*	*soror*	Afrotropic	Mozambique	23	93	KT892107	KT892399
200	Brsp.Plcy.5.30.2012.8	FMNH	*Brueelia*	*sp.*	Platysteiridae	*Platysteira*	*cyanea*	Afrotropic	Mozambique	23	93	KT892240	KT892530

Table#	Louse voucher	Deposition of lice*	Louse Genus	Louse species	Host family	Host Genus	Host species	BioRegion	Country	5%OTU (n = 166 OTUs)	bGMYC OTU (n = 114 OTUs)	COI Accestion#	EF-1a Accession#
201	Brsp.Maru.1.25.2011.12	FMNH	*Brueelia*	*sp.*	Ploceidae	*Anaplectes*	*rubriceps*	Afrotropic	Malawi	153	96	KT892194	KT892486
202	Brsp.Eual.1.25.2011.9	FMNH	*Brueelia*	*sp.*	Ploceidae	*Euplectes*	*albonotatus*	Afrotropic	Malawi	124	20	KT892164	KT892456
203	Brsp.Euar.1.25.2011.5	FMNH	*Brueelia*	*sp.*	Ploceidae	*Euplectes*	*ardens*	Afrotropic	Malawi	45	65	KT892165	KT892457
204	Brsp.Euar.5.30.2012.2	FMNH	*Brueelia*	*sp.*	Ploceidae	*Euplectes*	*ardens*	Afrotropic	Mozambique	45	65	KT892166	KT892458
205	Brsp.Manit.4.16.2012.8	INHS	*Brueelia*	*sp.*	Ploceidae	*Malimbus*	*nitens*	Afrotropic	Ghana	4	112	KT892193	KT892485
206	Brsp.Plru.1.25.2011.3	FMNH	*Brueelia*	*sp.*	Passeridae	*Plocepasser*	*rufoscapulatus*	Afrotropic	Malawi	46	90	KT892241	KT892531
207	Brsp.Plru.11.15.2010.17	FMNH	*Brueelia*	*sp.*	Passeridae	*Plocepasser*	*rufoscapulatus*	Afrotropic	Malawi	46	90	KT892242	KT892532
208	Brsp.Plcu.11.15.2010.11	FMNH	*Brueelia*	*sp.*	Ploceidae	*Ploceus*	*cucullatus*	Afrotropic	Malawi	69	29	KT892239	KT892529
209	Stsp.Plvel.2.3.1999.3	INHS	*Brueelia*	*sp.*	Ploceidae	*Ploceus*	*velatus*	Afrotropic	South Africa	81	96	AY149392	AY149422
210	Brsp.Ququ.11.15.2010.15	FMNH	*Brueelia*	*queleae*	Ploceidae	*Quelea*	*quelea*	Afrotropic	Malawi	116	32	KT892255	KT892545
211	Brsp.Aicra.8.31.2011.2	PIPeR	*Brueelia*	*sp.*	Ptilonorhynchidae	*Ailuroedus*	*crassirostris*	Australasia	Australia	2	114	KT892091	KT892383
212	Brsp.Aicra.9.6.2011.1	PIPeR	*Brueelia*	*sp.*	Ptilonorhynchidae	*Ailuroedus*	*crassirostris*	Australasia	Australia	2	114	KT892092	KT892384
213	Brsp.Chnuc.05.30.2012.06	PIPeR	*Brueelia*	*sp.*	Ptilonorhynchidae	*Chlamydera*	*nuchalis*	Australasia	Australia	2	114	KT892118	KT892410
214	Brsp.Chnuc.05.30.2012.07	PIPeR	*Brueelia*	*sp.*	Ptilonorhynchidae	*Chlamydera*	*nuchalis*	Australasia	Australia	2	114	KT892119	KT892411
215	Brsp.Chnuc.11.28.2011.10	PIPeR	*Brueelia*	*sp.*	Ptilonorhynchidae	*Chlamydera*	*nuchalis*	Australasia	Australia	2	114	KT892120	KT892412
216	Brsp.Chnuc.12.13.2011.6	PIPeR	*Brueelia*	*sp.*	Ptilonorhynchidae	*Chlamydera*	*nuchalis*	Australasia	Australia	2	114	KT892121	KT892413
217	Brsp.Angra.11.8.2011.7	INHS	*Brueelia*	*sp.*	Pycnonotidae	*Stelgidillas*	*gracilirostris*	Afrotropic	Ghana	4	112	KT892098	KT892390
218	Brsp.Anlat.4.16.2012.4	INHS	*Brueelia*	*sp.*	Pycnonotidae	*Eurillas*	*latirostris*	Afrotropic	Ghana	4	112	KT892099	KT892391
219	Brsp.Anmi.4.5.2011.5	FMNH	*Brueelia*	*sp.*	Pycnonotidae	*Arizelocichla*	*milanjensis*	Afrotropic	Malawi	9	112	KT892100	KT892392
220	Brsp.Anvi.4.5.2011.12	FMNH	*Brueelia*	*sp.*	Pycnonotidae	*Eurillas*	*virens*	Afrotropic	Malawi	41	59	KT892101	KT892393
221	Brsp.Blcan.9.6.2011.12	INHS	*Brueelia*	*sp.*	Pycnonotidae	*Bleda*	*canicapillus*	Afrotropic	Ghana	4	112	KT892109	KT892401
222	Brsp.Blexi.8.31.2011.9	INHS	*Brueelia*	*sp.*	Pycnonotidae	*Bleda*	*eximius*	Afrotropic	Ghana	4	112	KT892110	KT892402
223	Brsp.Blsyn.11.8.2011.5	INHS	*Brueelia*	*sp.*	Pycnonotidae	*Bleda*	*syndactylus*	Afrotropic	Ghana	4	112	KT892111	KT892403
224	Brsp.Crbar.11.8.2011.4	INHS	*Brueelia*	*sp.*	Pycnonotidae	*Criniger*	*barbatus*	Afrotropic	Ghana	4	112	KT892145	KT892437
225	Brsp.Hecas.12.11.2011.4	PIPeR	*Brueelia*	*sp.*	Pycnonotidae	*Hemixos*	*castanonotus*	Indo-Malayan	China	10	102	KT892176	KT892468
226	Brsp.Hyphi.10.25.2011.11	PIPeR	*Brueelia*	*sp.*	Pycnonotidae	*Hypsipetes*	*philippinus*	Indo-Malayan	Philippines	156	103	KT892179	KT892471
227	Brsp.Hyphi.7.14.1999.7	PIPeR	*Brueelia*	*sp.*	Pycnonotidae	*Hypsipetes*	*philippinus*	Indo-Malayan	Philippines	28	103	AY149385	AY149415
228	Brsp.Hypleu.12.11.2011.5	PIPeR	*Brueelia*	*sp.*	Pycnonotidae	*Hypsipetes*	*leucocephalus*	Indo-Malayan	China	28	103	KT892180	KT892472
229	Brsp.Hypmcc.12.11.2011.6	PIPeR	*Brueelia*	*sp.*	Pycnonotidae	*Ixos*	*mcclellandii*	Indo-Malayan	China	10	102	KT892181	KT892473
230	BrspHyleu415201211	PIPeR	*Brueelia*	*sp.*	Pycnonotidae	*Hypsipetes*	*leucocephalus*	Indo-Malayan	China	28	103	KT892299	KT892589
231	Brsp.Phalb.9.6.2011.11	INHS	*Brueelia*	*sp.*	Pycnonotidae	*Phyllastrephus*	*albigularis*	Afrotropic	Ghana	4	112	KT892234	KT892524
232	Brsp.Phfl.1.25.2011.15	FMNH	*Brueelia*	*sp.*	Pycnonotidae	*Phyllastrephus*	*flavostriatus*	Afrotropic	Malawi	67	112	KT892235	KT892525
233	Brsp.Phfl.2.9.2011.27	FMNH	*Brueelia*	*sp.*	Pycnonotidae	*Phyllastrephus*	*flavostriatus*	Afrotropic	Malawi	9	112	KT892236	KT892526
234	Brsp.Phict.4.16.2012.5	INHS	*Brueelia*	*sp.*	Pycnonotidae	*Phyllastrephus*	*icterinus*	Afrotropic	Ghana	4	112	KT892237	KT892527
235	Brsp.Pyba.2.9.2011.39	FMNH	*Brueelia*	*sp.*	Pycnonotidae	*Pycnonotus*	*barbatus*	Afrotropic	Malawi	120	71	KT892249	KT892539
236	Brsp.Pygoa.11.8.2011.10	PIPeR	*Brueelia*	*sp.*	Pycnonotidae	*Pycnonotus*	*goiavier*	Indo-Malayan	Malaysia	43	72	KT892250	KT892540
237	Brsp.Pynig.1.12.1999.8	–	*Brueelia*	*sp.*	Pycnonotidae	*Pycnonotus*	*nigricans*	Afrotropic	South Africa	119	71	AY149397	AY149427
238	Brsp.Pyplu.11.8.2011.9	PIPeR	*Brueelia*	*sp.*	Pycnonotidae	*Pycnonotus*	*plumosus*	Indo-Malayan	Malaysia	43	72	KT892252	KT892542
239	Brsp.Pyxan.12.11.2011.13	PIPeR	*Brueelia*	*sp.*	Pycnonotidae	*Pycnonotus*	*xanthorrhous*	Indo-Malayan	China	10	102	KT892253	KT892543
240	Brsp.Sphsp.12.11.2011.14	PIPeR	*Brueelia*	*sp.*	Pycnonotidae	*Spizixos*	*semitorques*	Indo-Malayan	China	10	102	KT892262	KT892552
241	Brla.9.14.2011.9	FMNH	*Traihoriella*	*laticeps*	Ramphastidae	*Andigena*	*nigrirostris*	Neotropic	Peru	6	110	KT892080	KT892372

Table#	Louse voucher	Deposition of lice*	Louse Genus	Louse species	Host family	Host Genus	Host species	BioRegion	Country	5%OTU (n = 166 OTUs)	bGMYC OTU (n = 114 OTUs)	COI Accession#	EF-1a Accession#
242	Brla.Anni.9.28.2011.1	FMNH	*Traihoriella*	*laticeps*	Ramphastidae	*Andigena*	*nigrirostris*	Neotropic	Peru	6	110	KT892081	KT892373
243	Brlat.1.17.2000.14	FMNH	*Traihoriella*	*laticeps*	Ramphastidae	*Andigena*	*nigrirostris*	Neotropic	Peru	6	110	AY149398	AY149428
244	Brla.Aulco.9.28.2011.10	FMNH	*Traihoriella*	*laticeps*	Ramphastidae	*Aulacorhynchus*	*coeruleicinctis*	Neotropic	Bolivia	131	110	KT892082	KT892374
245	Brla.Aulpr.9.28.2011.6	FMNH	*Traihoriella*	*laticeps*	Ramphastidae	*Aulacorhynchus*	*prasinus*	Neotropic	Peru	6	110	KT892083	KT892375
246	Brlat.1.17.2000.15	FMNH	*Traihoriella*	*laticeps*	Ramphastidae	*Aulacorhynchus*	*prasinus*	Neotropic	Peru	6	110	AY149399	AY149429
247	Brsp.Aude.9.14.2011.10	FMNH	*Traihoriella*	*laticeps*	Ramphastidae	*Aulacorhynchus*	*derbianus*	Neotropic	Peru	6	110	KT892103	KT892395
248	Brsp.Ausu.9.14.2011.12	FMNH	*Traihoriella*	*laticeps*	Ramphastidae	*Aulacorhynchus*	*sulcatus*	Neotropic	Venezuela	6	110	KT892104	KT892396
249	Brsp.Sifro.7.14.1999.1	PIPeR	*Brueelia*	*sp.*	Sittidae	*Sitta*	*frontalis*	Indo-Malayan	Philippines	1	114	AY149383	AY149413
250	Brsp.Cucey.10.4.2011.12	PIPeR	*Brueelia*	*sp.*	Stenostiridae	*Culicicapa*	*ceylonensis*	Indo-Malayan	China	135	114	KT892149	KT892441
251	Brsp.Lapur.8.31.2011.7	INHS	*Brueelia*	*sp.*	Sturnidae	*Lamprotornis*	*purpureus*	Afrotropic	Ghana	44	67	KT892186	KT892478
252	Brsp.Negu.11.29.2010.20	FMNH	*Brueelia*	*sp.*	Sturnidae	*Neocichla*	*gutturalis*	Afrotropic	Malawi	79	25	KT892211	KT892501
253	Brsp.Onte.4.5.2011.10	FMNH	*Brueelia*	*sp.*	Sturnidae	*Onychognathus*	*tenuirostris*	Afrotropic	Malawi	78	26	KT892214	KT892504
254	Brneb.Stvul.1.23.2012.2	PIPeR	*Brueelia*	*sp.*	Sturnidae	*Sturnus*	*vulgaris*	Palearctic	Sweden	138	9	KT892084	KT892376
255	Brsp.Bema.4.5.2011.20	FMNH	*Brueelia*	*sp.*	Bernieridae	*Bernieria*	*madagascariensis*	Afrotropic	Madagascar	33	60	KT892108	KT892400
256	Brsp.Brci.4.5.2011.1	FMNH	*Brueelia*	*sp.*	Locustellidae	*Bradypterus*	*cinnamomeus*	Afrotropic	Malawi	1	114	KT892112	KT892404
257	Brsp.Chsi.4.5.2011.13	FMNH	*Brueelia*	*sp.*	Acrocephalidae	*Iduna*	*similis*	Afrotropic	Malawi	1	114	KT892123	KT892415
258	BrspPagul42020121	PIPeR	*Brueelia*	*sp.*	Paradoxornithidae	*Paradoxornis*	*gularis*	Indo-Malayan	China	63	75	KT892312	KT892602
259	BrspPagul42020122	PIPeR	*Brueelia*	*sp.*	Paradoxornithidae	*Paradoxornis*	*gularis*	Indo-Malayan	China	63	75	KT892313	KT892603
260	Brsp.Pasub.2.3.1999.5	FMNH	*Brueelia*	*sp.*	Sylviidae	*Sylvia*	*subcaeruleum*	Afrotropic	South Africa	65	28	AY149396	AY149426
261	Brsp.Pyino.12.11.2011.11	PIPeR	*Brueelia*	*sp.*	Phylloscopidae	*Phylloscopus*	*inornatus*	Indo-Malayan	China	1	114	KT892251	KT892541
262	Gennov.Raps.8.25.2011.3	FMNH	*Brueelia*	*sp.*	Bernieridae	*Randia*	*pseudozosterops*	Afrotropic	Madagascar	118	47	KT892334	KT892624
263	Brsp.Xaci.4.5.2011.21	FMNH	*Brueelia*	*sp.*	Bernieridae	*Xanthomixis*	*cinereiceps*	Afrotropic	Madagascar	33	60	KT892276	KT892566
264	Brsp.Almor.10.4.2011.10	PIPeR	*Brueelia*	*sp.*	Leiothrichidae	*Alcippe*	*morrisonia*	Indo-Malayan	China	53	58	KT892094	KT892386
265	Brsp.Almor.10.4.2011.9	PIPeR	*Brueelia*	*sp.*	Leiothrichidae	*Alcippe*	*morrisonia*	Indo-Malayan	China	53	58	KT892095	KT892387
266	Brsp.Gacin.10.4.2011.15	PIPeR	*Brueelia*	*sp.*	Leiothrichidae	*Lanthocincla*	*cineracea*	Indo-Malayan	China	121	66	KT892170	KT892462
267	Brsp.Gamae.10.4.2011.5	PIPeR	*Brueelia*	*sp.*	Leiothrichidae	*Garrulax*	*maesi*	Indo-Malayan	China	163	85	KT892171	KT892463
268	BrspGamae415201239	PIPeR	*Brueelia*	*sp.*	Leiothrichidae	*Garrulax*	*maesi*	Indo-Malayan	China	90	85	KT892297	KT892587
269	BrspGamil32620124A	PIPeR	*Brueelia*	*sp.*	Leiothrichidae	*Trochalopteron*	*milnei*	Indo-Malayan	China	92	38	KT892298	KT892588
270	BrspLipho420201225	PIPeR	*Brueelia*	*sp.*	Leiothrichidae	*Liocichla*	*phoenicea*	Indo-Malayan	China	56	105	KT892301	KT892591
271	Brsp.Narab.10.25.2011.5	–	*Brueelia*	*sp.*	Pellorneidae	*Robsonius*	*rabori*	Indo-Malayan	Philippines	80	69	KT892210	KT892500
272	BrspNabre415201221	PIPeR	*Brueelia*	*sp.*	Pellorneidae	*Turdinus*	*brevicaudatus*	Indo-Malayan	China	96	69	KT892309	KT892599
273	BrspNabre415201222	PIPeR	*Brueelia*	*sp.*	Pellorneidae	*Turdinus*	*brevicaudatus*	Indo-Malayan	China	97	91	KT892310	KT892600
274	Brsp.Poruf.12.11.2011.12	PIPeR	*Brueelia*	*sp.*	Timaliidae	*Pomatorhinus*	*ruficollis*	Indo-Malayan	China	71	66	KT892243	KT892533
275	BrspPoruf326201234	–	*Brueelia*	*sp.*	Timaliidae	*Pomatorhinus*	*ruficollis*	Indo-Malayan	China	104	85	KT892314	KT892604
276	Brsp.Ststr.10.4.2011.7	PIPeR	*Brueelia*	*sp.*	Timaliidae	*Stachyris*	*striolata*	Indo-Malayan	China	38	91	KT892266	KT892556
277	Brsp.Ststr.10.4.2011.8	PIPeR	*Brueelia*	*sp.*	Timaliidae	*Stachyris*	*striolata*	Indo-Malayan	China	38	91	KT892267	KT892557
278	Bana.6.27.2006.18	FMNH	*Brueelia*	*anamariae*	Troglodytidae	*Troglodytes*	*aedon*	Nearctic	USA	161	113	FJ171220	FJ171243
279	Trsp.Trmas.1.12.1999.9	–	*Brueelia*	*sp.*	Trogonidae	*Trogon*	*massena*	Neotropic	Mexico	54	79	AY149386	AY149416
280	Trsp.Trmel.5.4.1999.5	PIPeR	*Brueelia*	*cicchinoi*	Trogonidae	*Trogon*	*melanocephalus*	Neotropic	Mexico	54	79	AY149387	AY149417
281	Brsp.Alfu.2.9.2011.31	FMNH	*Brueelia*	*sp.*	Muscicapidae	*Pseudalethe*	*fuelleborni*	Afrotropic	Malawi	1	114	KT892093	KT892385
282	Bran.6.13.2006.1	FMNH	*Brueelia*	*antiqua*	Turdidae	*Catharus*	*guttatus*	Nearctic	USA	3	111	FJ171221	FJ171244

Table#	Louse voucher	Deposition of lice*	Louse Genus	Louse species	Host family	Host Genus	Host species	BioRegion	Country	5%OTU (n = 166 OTUs)	bGMYC OTU (n = 114 OTUs)	COI Accestion#	EF-1a Accession#
283	Bran.6.27.2006.30	FMNH	*Brueelia*	*antiqua*	Turdidae	*Catharus*	*guttatus*	Nearctic	USA	3	111	FJ171222	FJ171245
284	Brsp.Cafr.6.13.2006.4	FMNH	*Sturnidoecus*	*antiqua*	Turdidae	*Catharus*	*fuscater*	Neotropic	Panama	17	94	FJ171225	FJ171248
285	Brsp.Cafu.6.13.2006.2	FMNH	*Brueelia*	*antiqua*	Turdidae	*Catharus*	*fuscescens*	Neotropic	Bolivia	3	111	FJ171227	FJ171250
286	Brsp.Cafu.6.13.2006.6	FMNH	*Brueelia*	*antiqua*	Turdidae	*Catharus*	*fuscescens*	Nearctic	USA	3	111	FJ171228	FJ171251
287	Brsp.Cafu.6.27.2006.31	FMNH	*Brueelia*	*antiqua*	Turdidae	*Catharus*	*fuscescens*	Nearctic	USA	3	111	FJ171229	FJ171252
288	Brsp.Cami.6.13.2006.5	FMNH	*Brueelia*	*antiqua*	Turdidae	*Catharus*	*minimus*	Nearctic	USA	3	111	KT892115	KT892407
289	Brze.6.13.2006.10	FMNH	*Brueelia*	*antiqua*	Turdidae	*Catharus*	*ustulatus*	Nearctic	USA	3	111	FJ171237	FJ171261
290	Brze.6.13.2006.3	FMNH	*Brueelia*	*antiqua*	Turdidae	*Catharus*	*ustulatus*	Neotropic	Panama	3	111	FJ171238	FJ171262
291	Brze.6.13.2006.7	FMNH	*Brueelia*	*antiqua*	Turdidae	*Catharus*	*ustulatus*	Nearctic	USA	3	111	FJ171239	FJ171263
292	Brze.6.27.2006.29	FMNH	*Brueelia*	*antiqua*	Turdidae	*Catharus*	*ustulatus*	Nearctic	USA	3	111	FJ171240	FJ171264
293	Brsp.Coalb.9.6.2011.8	FMNH	*Brueelia*	*sp.*	Muscicapidae	*Copsychus*	*albospecularis*	Afrotropic	Madagascar	142	100	KT892129	KT892421
294	Brsp.Mycol.11.28.2011.11	INHS	*Brueelia*	*sp.*	Turdidae	*Myadestes*	*coloratus*	Neotropic	Panama	49	57	KT892209	KT892499
295	Brsp.Nepoe.11.8.2011.3	INHS	*Brueelia*	*sp.*	Turdidae	*Neocossyphus*	*poensis*	Afrotropic	Ghana	4	112	KT892212	KT892502
296	BrspSicur326201227A	PIPeR	*Brueelia*	*sp.*	Turdidae	*Sialia*	*currucoides*	Nearctic	USA	105	42	KT892319	KT892609
297	Bril.5.30.2012.19	FMNH	*Brueelia*	*iliaci*	Turdidae	*Turdus*	*migratorius*	Nearctic	USA	55	81	KT892078	KT892370
298	Brneo.Tuobs.1.23.2012.5	PIPeR	*Sturnidoecus*	*sp.*	Turdidae	*Turdus*	*obscurus*	Palearctic	Japan	16	92	KT892085	KT892377
299	Brsp.Tuchr.1.23.2012.6	PIPeR	*Sturnidoecus*	*sp.*	Turdidae	*Turdus*	*chrysolaus*	Palearctic	Japan	16	92	KT892270	KT892560
300	Brsp.Zocit.12.13.2011.4	PIPeR	*Brueelia*	*sp.*	Turdidae	*Geokichla*	*citrina*	Indo-Malayan	China	36	104	KT892277	KT892567
301	Brsp.Zogu.4.5.2011.11	FMNH	*Brueelia*	*sp.*	Turdidae	*Geokichla*	*gurneyi*	Afrotropic	Malawi	98	36	KT892278	KT892568
302	Brsp.Zohei.9.6.2011.9	PIPeR	*Brueelia*	*sp.*	Turdidae	*Zoothera*	*heinei*	Australasia	Australia	12	99	KT892279	KT892569
303	Brsp.Zolun.05.30.2012.08	PIPeR	*Brueelia*	*sp.*	Turdidae	*Zoothera*	*lunulata*	Australasia	Australia	12	99	KT892280	KT892570
304	Brsp.Zolun.8.31.2011.6	PIPeR	*Brueelia*	*sp.*	Turdidae	*Zoothera*	*lunulata*	Australasia	Australia	12	99	KT892281	KT892571
305	Brsp.Zolun.9.6.2011.2	PIPeR	*Brueelia*	*sp.*	Turdidae	*Zoothera*	*lunulata*	Australasia	Australia	12	99	KT892282	KT892572
306	BrspZocit326201236	PIPeR	*Sturnidoecus*	*sp.*	Turdidae	*Zoothera*	*citrina*	Indo-Malayan	China	16	92	KT892326	KT892616
307	BrspZodau121320115	PIPeR	*Sturnidoecus*	*sp.*	Turdidae	*Zoothera*	*dauma*	Indo-Malayan	China	109	43	KT892327	KT892617
308	Brsp.Vacur.9.6.2011.7	INHS	*Brueelia*	*sp.*	Vangidae	*Vanga*	*curvirostris*	Afrotropic	Madagascar	9	112	KT892273	KT892563
309	Brsp.Vima.4.5.2011.4	FMNH	*Brueelia*	*sp.*	Viduidae	*Vidua*	*macroura*	Afrotropic	Malawi	101	34	KT892274	KT892564
310	Brsp.Zose.2.9.2011.36	FMNH	*Brueelia*	*sp.*	Zosteropidae	*Zosterops*	*senegalensis*	Afrotropic	Malawi	42	73	KT892283	KT892573
311	Blsp.Brlep.2.4.2002.10	INHS	*Buerelius*	*longiceps*	Brachypteraciidae	*Brachypteracias*	*leptosomus*	Afrotropic	Madagascar	151	2	KT892067	KT892359
312	Stsp.Mabl.12.21.2011.11	FMNH	*Sturnidoecus*	*sp.*	Malaconotidae	*Malaconotus*	*blanchoti*	Afrotropic	Malawi	60	78	KT892348	KT892638
313	Stsp.Eu.5.30.2012.11	FMNH	*Sturnidoecus*	*sp.*	Ploceidae	*Euplectes*	*ardens*	Afrotropic	Mozambique	11	106	KT892342	KT892632
314	Stsp.Eual.12.21.2011.6	FMNH	*Sturnidoecus*	*sp.*	Ploceidae	*Euplectes*	*albonotatus*	Afrotropic	Malawi	11	106	KT892343	KT892633
315	Stsp.Eual.12.21.2011.7	FMNH	*Sturnidoecus*	*sp.*	Ploceidae	*Euplectes*	*albonotatus*	Afrotropic	Malawi	11	106	KT892344	KT892634
316	Stsp.Euar.12.21.2011.8	FMNH	*Sturnidoecus*	*sp.*	Ploceidae	*Euplectes*	*ardens*	Afrotropic	Malawi	11	106	KT892345	KT892635
317	Stsp.Euar.5.30.2012.12	FMNH	*Sturnidoecus*	*sp.*	Ploceidae	*Euplectes*	*ardens*	Afrotropic	Mozambique	11	106	KT892346	KT892636
318	Stsp.Ploc.5.30.2012.10	FMNH	*Sturnidoecus*	*sp.*	Ploceidae	*Ploceus*	*ocularis*	Afrotropic	Mozambique	62	107	KT892350	KT892640
319	Stsp.Ploc.5.30.2012.9	FMNH	*Sturnidoecus*	*sp.*	Ploceidae	*Ploceus*	*ocularis*	Afrotropic	Mozambique	62	107	KT892351	KT892641
320	Stsp.Plve.12.21.2011.19	FMNH	*Sturnidoecus*	*sp.*	Ploceidae	*Ploceus*	*velatus*	Afrotropic	Malawi	58	107	KT892352	KT892642
321	Stxa.12.21.2011.20	FMNH	*Sturnidoecus*	*xanthops*	Ploceidae	*Ploceus*	*xanthops*	Afrotropic	Malawi	58	107	KT892355	KT892645
322	Stsp.Ququ.12.21.2011.21	FMNH	*Sturnidoecus*	*sp.*	Ploceidae	*Quelea*	*quelea*	Afrotropic	Malawi	82	107	KT892353	KT892643
323	Stsp.Cile.12.21.2011.3	FMNH	*Sturnidoecus*	*sp.*	Sturnidae	*Cinnyricinclus*	*leucogaster*	Afrotropic	Malawi	75	53	KT892341	KT892631

Table#	Louse voucher	Deposition of lice*	Louse Genus	Louse species	Host family	Host Genus	Host species	BioRegion	Country	5%OTU (n = 166 OTUs)	bGMYC OTU (n = 114 OTUs)	COI Accestion#	EF-1a Accession#
324	Snsp.Lapur.8.31.2011.8	FMNH	*Sturnidoecus*	*sp.*	Sturnidae	*Lamprotornis*	*purpureus*	Afrotropic	Ghana	70	50	KT892337	KT892627
325	Stsp.Lach.12.21.2011.10	FMNH	*Sturnidoecus*	*sp.*	Sturnidae	*Lamprotornis*	*chloropterus*	Afrotropic	Malawi	61	77	KT892347	KT892637
326	Stsp.Negu.12.21.2011.13	FMNH	*Sturnidoecus*	*sp.*	Sturnidae	*Neocichla*	*gutturalis*	Afrotropic	Malawi	61	77	KT892349	KT892639
327	Stsp.Hymu.6.13.2006.11	FMNH	*Sturnidoecus*	*sp.*	Turdidae	*Hylocichla*	*mustelina*	Nearctic	USA	17	94	FJ171241	FJ171265
328	Stsp.Hymu.6.13.2006.8	FMNH	*Sturnidoecus*	*sp.*	Turdidae	*Hylocichla*	*mustelina*	Nearctic	USA	17	94	FJ171242	FJ171266
329	Snsp.Tugra.10.16.2002.1	INHS	*Sturnidoecus*	*caligineus*	Turdidae	*Turdus*	*grayi*	Neotropic	Costa Rica	17	94	JX121680	JX121694
330	Snsp.Tupel.4.16.2012.12	INHS	*Sturnidoecus*	*sp.*	Turdidae	*Turdus*	*pelios*	Afrotropic	Ghana	68	51	KT892338	KT892628
331	Stsi.12.21.2011.25	PIPeR	*Sturnidoecus*	*simplex*	Turdidae	*Turdus*	*migratorius*	Nearctic	USA	66	52	KT892340	KT892630
332	Stsp.Tuli.12.21.2011.24	FMNH	*Sturnidoecus*	*sp.*	Turdidae	*Turdus*	*libonyanus*	Afrotropic	Malawi	60	78	KT892354	KT892644
333	Snsp.Zolat.11.8.2011.11	PIPeR	*Sturnidoecus*	*sp.*	Turdidae	*Geokichla*	*interpres*	Indo-Malayan	Malaysia	16	92	KT892339	KT892629
334	Alsp.Hamal.1.16.2001.11	–	*Alcedoecus*	*sp.*	Alcidinidae	*Halcyon*	*malimbica*	Afrotropic	Ghana	UP	OUTGROUP	KT892064	KT892356
335	Afdup.3.16.2001.10	–	*Alcedoffula*	*duplicata*	Alcedinidae	*Corythornis*	*leucogaster*	Afrotropic	Uganda	UP	OUTGROUP	JX121669	JX121682
336	Ausub.1.27.1999.12	PIPeR	*Austrophilopterus*	*sp.*	Ramphastidae	*Ramphastos*	*sulfuratus*	Neotropic	Mexico	UP	OUTGROUP	AF444850	AF447188
337	Cabid.6.29.1998.2	–	*Campanulotes*	*compar*	Columbidae	*Columba*	*livia*	Nearctic	USA	UP	OUTGROUP	AF545681	AF278671
338	Chsp.Orcan.11.10.2001.9	–	*Chelopistes*	*sp.*	Cracidae	*Ortalis*	*canicollis*	Neotropic	Bolivia	UP	OUTGROUP	JX121674	JX121687
339	Dgcar.9.8.1999.7	PIPeR	*Degeeriella*	*carruthi*	Falconidae	*Falco*	*sparverius*	Nearctic	USA	UP	OUTGROUP	AF444860	AF447196
340	Embra.2.4.2002.11	–	*Emersoniella*	*bracteata*	Alcedinidae	*Dacelo*	*novaeguinnea*	Australasia	Australia	UP	OUTGROUP	KT892333	KT892623
341	Ffpal.11.22.2001.14	–	*Forficuloecus*	*palmai*	Psittacidae	*Barnardius*	*zonarius*	Australasia	Australia	UP	OUTGROUP	EU669828	JX121688
342	Fosp.Thdol.4.7.1999.10	PIPeR	*Formicaphagus*	*sp.*	Thamnophilidae	*Thamnophilus*	*doliatus*	Neotropic	Mexico	UP	OUTGROUP	AY149403	AY149433
343	Foana.1.27.1999.7	PIPeR	*Formicaricola*	*analoides*	Formicariidae	*Formicarius*	*analis*	Neotropic	Mexico	UP	OUTGROUP	AY149402	AY149432
344	Gosp.Phcol.11.10.2001.2	–	*Goniocotes*	*sp.*	Phasianidae	*Phasianus*	*colchicus*	Nearctic	USA	UP	OUTGROUP	HQ332829	HQ332891
345	Mrsp.Megul.4.3.2000.11	PIPeR	*Meropoecus*	*sp.*	Meropidae	*Merops*	*gularis*	Afrotropic	Ghana	73	48	AF545729	AF545792
346	Nseos.11.22.2001.15	–	*Neopsittaconirmus*	*eos*	Psittacidae	*Eolophus*	*roseicapilla*	Australasia	Australia	UP	OUTGROUP	KT892335	KT892625
347	Nylon.2.6.1999.6	PIPeR	*Nyctibicola*	*longirostris*	Nyctibiidae	*Nyctibius*	*jamaicensis*	Neotropic	Mexico	UP	OUTGROUP	AF444864	AF447199
348	Oscur.10.5.1999.2	PIPeR	*Osculotes*	*curta*	Opisthocomidae	*Opisthocomus*	*hoazin*	Neotropic	Brazil	UP	OUTGROUP	AF545737	AF348660
349	Oxchi.1.27.1999.6	PIPeR	*Oxylipeurus*	*chiniri*	Cracidae	*Ortalis*	*vetula*	Neotropic	Mexico	UP	OUTGROUP	AF545739	AF385025
350	Pasp.Arast.2.10.1999.7	PIPeR	*Paragoniocotes*	*sp.*	Laridae	*Aratinga*	*astec*	Neotropic	Mexico	UP	OUTGROUP	AY149404	AY149434
351	Brsp.Psmin.2.1.2000.9	PIPeR	*Penenirmus*	*sp.*	Aegithalidae	*Psaltriparus*	*minimus*	Nearctic	USA	UP	OUTGROUP	AY149409	AY149438
352	Pezum.1.12.1999.10	–	*Penenirmus*	*zumpti*	Ramphastidae	*Lybius*	*torquatus*	Afrotropic	South Africa	UP	OUTGROUP	AF444865	AF447200
353	Wiabs.10.5.1999.1	PIPeR	*Pessoaiella*	*absita*	Opisthocomidae	*Opisthocomus*	*hoazin*	Neotropic	Brazil	UP	OUTGROUP	JX121681	JX121695
354	Ppsp.Sppus.11.10.2001.11	–	*Philopterus*	*sp.*	Emberizidae	*Spizella*	*pusilla*	Nearctic	USA	UP	OUTGROUP	AY314820	AY314841
355	Phcub.9.29.1998.7	–	*Physconelloides*	*cubanus*	Columbidae	*Geotrygon*	*montana*	Neotropic	Mexico	UP	OUTGROUP	AY149409	AY149439
356	Pclau.11.22.2001.13	–	*Psittaconirmus*	*launceloti*	Psittacidae	*Trichoglossus*	*haematodus*	Australasia	Australia	UP	OUTGROUP	KT892336	KT892626
357	Qkeos.5.16.2002.5	–	*Psittoecus*	*eos*	Psittacidae	*Cacatua*	*sanguinea*	Australasia	Australia	UP	OUTGROUP	JX121677	JX121691
358	Qupun.2.3.1999.2	PIPeR	*Quadraceps*	*punctatus*	Laridae	*Chroicocephalus*	*cirrocephalus*	Afrotropic	South Africa	UP	OUTGROUP	AY149405	JX121692
359	Raful.2.6.1999.11	PIPeR	*Rallicola*	*fuliginosa*	Dendrocolaptidae	*Dendrocincla*	*anabatina*	Neotropic	Mexico	UP	OUTGROUP	AY149408	AY149437
360	Racol.1.27.1999.2	PIPeR	*Rallicola*	*colombiana*	Dendrocolaptidae	*Dendrocolaptes*	*certhia*	Neotropic	Mexico	UP	OUTGROUP	AY149407	AY149436
361	Salar.4.7.1999.12	–	*Saemundssonia*	*lari*	Laridae	*Chroicocephalus*	*cirrocephalus*	Afrotropic	South Africa	UP	OUTGROUP	AY149406	AY149435
362	Sgorb.11.10.2001.10	PIPeR	*Strongylocotes*	*orbicularis*	Tinamidae	*Crypturellus*	*parvirostris*	Neotropic	Bolivia	UP	OUTGROUP	HQ332847	HQ332918
363	Tssp.Psbre.10.16.2002.6	–	*Theresiella*	*sp.*	Psittacidae	*Psittacella*	*brehmii*	Australasia	[illegible] Guinea	UP	OUTGROUP	EU669831	KT892646
364	Veber.10.17.2000.7	INHS	*Vernoniella*	*bergi*	Cuculidae	*Guira*	*guira*	Neotropic	Brazil	UP	OUTGROUP	AY314824	AY314844
